# An Intergenic Region Shared by *At4g35985* and *At4g35987* in *Arabidopsis thaliana* Is a Tissue Specific and Stress Inducible Bidirectional Promoter Analyzed in Transgenic Arabidopsis and Tobacco Plants

**DOI:** 10.1371/journal.pone.0079622

**Published:** 2013-11-19

**Authors:** Joydeep Banerjee, Dipak Kumar Sahoo, Nrisingha Dey, Robert L. Houtz, Indu Bhushan Maiti

**Affiliations:** 1 KTRDC, College of Agriculture, Food and Environment, University of Kentucky, Lexington, Kentucky, United States of America; 2 Department of Horticulture and Plant Physiol/Biochemistry/Molecular Biology Program, University of Kentucky, Lexington, Kentucky, United States of America; 3 Department of Gene Function and Regulation, Institute of Life Sciences, Bhubaneswar, Orissa, India; University of Delhi South Campus, India

## Abstract

On chromosome 4 in the *Arabidopsis* genome, two neighboring genes (calmodulin methyl transferase *At4g35987* and senescence associated gene *At4g35985*) are located in a head-to-head divergent orientation sharing a putative bidirectional promoter. This 1258 bp intergenic region contains a number of environmental stress responsive and tissue specific *cis*-regulatory elements. Transcript analysis of *At4g35985* and *At4g35987* genes by quantitative real time PCR showed tissue specific and stress inducible expression profiles. We tested the bidirectional promoter-function of the intergenic region shared by the divergent genes *At4g35985* and *At4g35987* using two reporter genes (GFP and GUS) in both orientations in transient tobacco protoplast and Agro-infiltration assays, as well as in stably transformed transgenic Arabidopsis and tobacco plants. In transient assays with GFP and GUS reporter genes the *At4g35985* promoter (P85) showed stronger expression (about 3.5 fold) compared to the *At4g35987* promoter (P87). The tissue specific as well as stress responsive functional nature of the bidirectional promoter was evaluated in independent transgenic *Arabidopsis* and tobacco lines. Expression of P85 activity was detected in the midrib of leaves, leaf trichomes, apical meristemic regions, throughout the root, lateral roots and flowers. The expression of P87 was observed in leaf-tip, hydathodes, apical meristem, root tips, emerging lateral root tips, root stele region and in floral tissues. The bidirectional promoter in both orientations shows differential up-regulation (2.5 to 3 fold) under salt stress. Use of such regulatory elements of bidirectional promoters showing spatial and stress inducible promoter-functions in heterologous system might be an important tool for plant biotechnology and gene stacking applications.

## Introduction

The intergenic region between two adjacent genes located on opposite strands of DNA is generally considered as a ‘putative bidirectional’ promoter. The bioinformatic analysis and subsequent experimental studies of the available complete genome sequence of many eukaryotes (human, yeast, plants), invertebrates and vertebrates showed a genome-wide presence of bidirectional promoters [1]–[7]. From the human genome-wide analysis, the distance between two transcription start sites (TSS) of a bidirectional promoter for two adjacent divergent genes is commonly considered to be within 1000 base pairs apart [1]. However, plant genomes contain intergenic region with TSS of bidirectional promoters longer than 1 kb base pairs apart [8]. In the *Arabidopsis* genome a large proportion (13.3%) of bidirectional gene pairs are available and among them a certain percentage share an intergenic region of 1 to 2 kb [5]. Bidirectional promoters having unique regulation and expression pattern has been reported in a number of organisms including *Saccharomyces cerevisiae* [6], [9].

A number of bidirectional promoters have been identified in animals including chicken, mouse, rat and human [7], [10]. The first evidence of a head-to-head gene orientation was observed in the mouse DHFR gene [11]. Similarly, one of the earlier reports of a bidirectional nuclear gene promoter in plants was the oleosin gene promoter exhibiting spatial and temporal regulation as well as inducible expression in an orientation dependent manner [12].

The availability of plant genome sequence data and bioinformatics studies revealed that plants like rice, populus and *Arabidopsis* possess a number of bidirectional promoters in their genomes and the activity of these promoters were further characterized [5], [13], [14]. Bidirectional promoters from melon [15] and *Capsicum annuum* [16] were also reported. In hot pepper, the bidirectional promoter located between the *CaTin*1 and *CaTin*1–2 genes, regulates the expression of two genes during biotic stress from pathogen infection [16]. In *Arabidopsis thaliana*, it was documented that a bidirectional promoter with divergent genes *cab*1 and *cab*2 was equally strong in both orientations [8]. On the other hand, an intergenic region in *Arabidopsis* chromosome 5 located between locus At5g06290 (codes 2-Cys peroxiredoxin B) and locus At5g06280 (protein of unknown function) showed tissue specific and stress inducible bidirectional promoter activity that functioned in an orientation dependent manner [17].

Likewise researchers have generated naturally occurring bidirectional promoters and a number of engineered bidirectional promoters. Recently a vascular tissue specific plant bidirectional promoter was constructed using the *grp 1.8* promoter from Chinese bean and the 4CL1 promoter from *Populus tomentosa* [18]. A bidirectional promoter module derived from poplar PtDrl02 promoter gave methyl jasmonate inducible expression of genes [19]. Genetic elements of viral or bacterial origin have also been used for designing bidirectional promoters for expressing genes in plants [20]–[22]. For molecular biology and biotechnology applications, several constitutive promoters have been developed from para-retrovirus genomes like *Cauliflower mosaic virus* 35S and 19S promoter [23], [24], *Mirabilis Mosaic Virus* (MMV) full-length and subgenomic transcript promoters [25], [26] *Figwort mosaic virus* (FMV) full-length and subgenomic transcript promoters [27], [28] and *Peanut chlorotic streak virus* (PClSV) full-length transcript promoter [29], *Strawberry vein banding virus* (SVBV) full-length transcript promoter [30]. Other examples of genetic promoters including *Agrobacterium* T-DNA gene-based *nos* [31], [32] and *mas* [33] constitutive promoters; and plant-based constitutive promoters like maize ubiquitin gene promoter [34], rice ubiquitin gene promoter [35], [36], rice actin1 gene promoter [37] have been used for various plant modification and biotechnology applications. Tissue specific promoters like soybean (*Glycine max*) glycinin gene seed-specific promoter [38] and oil seed *(Brassica napus*) seed specific napin promoter [39] have been used for expressing gene products in a target specific manner. Additionally, inducible promoters including the light inducible rbcS-3A promoter from pea plant [40], ethylene inducible tobacco chitinase gene Chn48 promoter [41], heavy metal inducible PvSR2 promoter from bean (*Phaseolus vulgaris*) [42] have been characterized. In spite of using a number of unidirectional promoters, bidirectional promoter could be the better choice for improving quantitative characters of crop plants and trait stacking in plants, which may reduce gene-silencing effects. Plant genomes can regulate multiple genes involved in biological and biochemical pathways in an efficient manner using bidirectional promoters that utilize less energy particularly in activating the expression of multiple genes. Bidirectional promoters can be used for co-expressing multi-gene traits. They can also regulate co-expression of genes functioning in the same or related biological pathways. In addition, a natural bidirectional promoter will aid in the expression of a gene close to its physiological conditions. A bidirectional promoter can generate protein products from two adjacent related genes in stoichiometric quantities, which is biologically significant [15].

In the present study, we report isolation and characterization of a 1258 bp bidirectional promoter (P85-P87), an intergenic region (IR) shared by *Arabidopsis* At4g35985 and *At4g35987* divergent genes on chromosome 4. The bidirectional promoter showed orientation dependent tissue specific expression of reporter genes (GFP and GUS), analyzed in transgenic *Arabidopsis* and tobacco plants. A number of stress related *cis*-elements are present in the promoter region. The bidirectional promoter P85–P87 (containing *At4g35985* promoter and *At4g35987* promoter) was regulated by a number of abiotic as well as biotic stresses in native *Arabidopsis* and in heterologous tobacco systems. The bidirectional promoter P85–P87 could be useful for expressing tissue specific and stress inducible transgene in heterologous plant systems.

## Materials and Methods

### Construction of bidirectional promoter-reporter gene plasmids: pB-GFP::P85–P87::GUS and pB-GFP::P87–P85::GUS for transient expression analysis

The *At4g35985* promoter driving the *At4g35985* gene and the *At4g35987* promoter driving the *At4g35987* gene were designated as P85 and P87 respectively. The straight and flipped oriented bidirectional promoters (P85–P87 and P87–P85), reporter genes (GFP and GUS), and CaMV 35S terminator (35ST) were amplified by PCR using appropriately designed primers as described below.

The bidirectional promoter of general structure 5′-XhoI-P85–P87-SstI-3′ was isolated from the intergenic region (IR, 1258-bp, *Arabidopsis* genomic coordinates 17,033,863–17,035120 in chromosome 4) shared by *At4g35985* and *At4g35987* genes by PCR amplification using a forward primer (5′-d-GCGGGCctcgagTATCGCCGGT GATTTAGG-3′) with a *Xho*I restriction site (lowercase), and the reverse primer (5′-d-ATGCAGgagctcTGAAGAATCGTTATAGCT-3′) with a *Sst*I restriction sites (lowercase). The flipped oriented bidirectional promoter of general structure 5′-*Xho*I-P87-P85-*Sac*I-3′ was PCR amplified from the 1258 bp intergenic region (IR) using a forward primer (5′-d GCTTACctcgagTGAAGAATCGTTATAGCT-3′) with a *Xho*I restriction site (lowercase), and the reverse primer (5′-d- GCTTACgagctcTATCGCCGGTGATTTAGG-3′) with a *Sac*I restriction site (lowercase). Restriction sites were incorporated to facilitate cloning.

The enhanced cDNA GFP (S65T) sequence as 5′-*Xho*I-GFP-*Hin*dIII-3′ was PCR amplified using the forward (5′-d-GCGGGCctcgagATGGTTTCTAAGGGTGAG-3′) and reverse primers (5′-d-ATGCAGaagcttTCACTTGTAGAGTTCATC-3′) having *Xho*I and *Hin*dIII sites (lowercase), respectively. The cDNA GUS gene as 5′-SstI- GUS- XbaI-3′ was PCR amplified using a forward primer (5′-d-GCGGGCgagctcATGGGATTAC GTCCTGTAGAA-3′) with an *Sst*I restriction site (lowercase), and a reverse primer (5′-d-ATGCAGtctagaTCATTGTTTGCCTCCCTG-3′) with an *Xba*I restriction site (lowercase). The CaMV 35S terminator (5′-*Hin*dIII-35ST-*Eco*RI-3′) was amplified using a forward primer (5′-d-GCGGGCaagcttGATCTGTCGATCGACAAGTT-3′) with a *Hin*dIII restriction site (lowercase), and a reverse primer (5′-d-ATGCAGgaattc TAATTCGGGGGATCTGGA-3′) with an *Eco*RI restriction site (lowercase). Promoter-reporter gene constructs of general physical structure, 5′-*Eco*RI-35S Terminator- *Hin*dIII- GFP -*Xho*I – P85-P87- *Sst*I- GUS- *Xba*I- 3′rbcS terminator-ClaI-3′ were assembled using gel-purification following PCR amplified genetic elements: 5′-*Hin*dIII-35ST-*Eco*RI-3′, 5′-*Xho*I-GFP-*Hin*dIII-3′, 5′-*Xho*I-P85-P87-*Sst*I-3′, 5′-*Xho*I-P87-P85-*Sst*I-3′, 5′-*Sst*I-GUS-*Xba*I-3′ into the corresponding sites of pKYLX80 [43] to generate the plasmid pB-GFP::P85-P87::GUS and pB-GFP::P87-P85::GUS for transient expression assay in tobacco protoplasts. The DNA sequence integrity of all constructs was confirmed before further use.

### Construction of pK-GFP::P85–P87::GUS and pK-GFP::P87–P85::GUS

For stable transformation in tobacco and *Arabidopsis*, fragments of general structure 5′-*Eco*RI-35ST-*Hin*dIII-GFP-*Xho*I-P85-P87-*Sst*I-GUS-*Xba*I-3′ and 5′-*Eco*RI-35ST-*Hin*dIII-GFP-*Xho*I-P87-P85-*Sst*I-GUS-*Xba*I-3′ were generated from pB-GFP::P85-P87::GUS and pB-GFP::P87-P85::GUS by PCR amplification using forward (5′-d-ATG CAGgaattcTAATTCGGGGGATCTGGA-3′) with an *Eco*RI restriction site (lowercase) and a reverse primer (5′-d-ATGCAGtctagaTCATTGTTT GCCTCCCTG-3′) with a *Xba*I restriction site (lowercase). Gel-purified PCR amplified fragments 5′-*Eco*RI-35ST-*Hin*dIII-GFP-*Xho*I-P85-P87-*Sst*I-GUS-*Xba*I-3′ and 5′-*Eco*RI-35ST-*Hin*dIII-GFP-*Xho*I-P87-P85-*Sst*I-GUS-*Xba*I-3′ were cloned separately into the corresponding sites of plant expression vector pKYLX71 [44] to generate plasmid pK-GFP::P85–P87::GUS and pK-GFP::P87–P85::GUS for transient Agro-infiltration assay in *Nicotiana benthamiana* and stable transformation of tobacco and *Arabidopsis*. The DNA sequence integrity of all constructs was confirmed before further use.

### Plant materials, growth condition and plant transformation

Generation of transgenic *Arabidopsis* plants (*Arabidopsis thaliana* ecotype Columbia-0) and tobacco transgenic lines (*Nicotiana tabacum* cv Samsun NN) and their maintenance were as described previously [30], [45]. The promoter-reporter gene constructs pB-GFP::P85-P87::GUS and pB-GFP::P87–P85::GUS with pKYLX80 vector were used for transient expression in tobacco Xanthi ‘Brad’ protoplast whereas pK-GFP::P85–P87::GUS and pK-GFP::P87–P85::GUS with pKYLX71-based vector were used for transient GUS expression in *Nicotiana benthamiana* leaf, and stable expression in *Arabidopsis* and tobacco plants. All pKYLX71 based constructs were mobilized into *Agrobacterium tumefaciens* GV3850 strain for Agro-infiltration and stable transformation as described previously [46]. Three-weeks-old *Arabidopsis* seedlings were used for obtaining young leaf and root tissue materials for extracting RNA and protein samples whereas for samples of older leaf, stem and flower tissues, 35–40 days old *Arabidopsis* plants grown in pots were used.

### RNA isolation and quantitative real-time PCR (qRT-PCR)

Total RNA was extracted from different plant tissues using the Plant RNeasy extraction kit (QIAGEN, CA, USA) following the manufacture' instruction. For quantitative measurement of *At4g35985*-specific transcript following forward primer (5′-d-ATGGAATGCTCTGCAACTCCTCCCAAGCTT-3′) and reverse primer (5′-d-GCAAGCGAGCTCTACGCTGTAGGATTTGTC-3′) were used. For quantitative measurement of the *At4g35987*-specific transcript, forward primer (5′-d-ATGGATCCCACTTCTTCTTCTTCCTCTGCT-3′), and reverse primer (5′-d-CTCGAAGTCATTGAGATCACTACAATTAT-3′) were used. For relative quantification of GUS specific transcript following primers were used: Forward primer 5′-d-TTACGTCCTGTAGAAACCCCA-3′ and reverse primer 5′-d-ACTGCCTGGCACAGCAATTGC-3′. The relative transcript abundance by real-time PCR was carried out following published protocol [47]. The PCR reaction was performed with four replicates and it was repeated with three biological samples. The transcript levels were measured following comparative Ct method (Applied Biosystems bulletin). For normalizing the amount of total RNA in all *Arabidopsis* samples, actin gene specific forward 5′-d-CTTGCACCAAGCAGCATGAA-3′ and reverse 5′-d-CCGATCCAGACACTGTACTTCCTT-3′ primers were used [48] whereas for tobacco samples α-tubulin gene specific forward 5′-d-ATGAGAGAGTGCATATCGAT-3′ and reverse 5′-d-TTCACTGAAGAAGGTGTTGAA-3′ primers were used [46].

### Transient expression assays: Protoplast electroporation and Agro-infiltration

Isolation of tobacco protoplasts from suspension cell cultures of *Nicotiana tabacum* L. cv Xanthi –Brad and electroporation of tobacco protoplasts with supercoiled plasmid DNA of promoter-reporter gene constructs were performed essentially as described earlier [27]. Transient Agro-infiltration assay was performed in *Nicotiana benthamiana* leaves using *A. tumefaciens* strain pGV3850 bearing pKYLX71 based binary vector promoter-reporter gene constructs following a previously published procedure [49].

### β-Glucuronidase (GUS) assay and histochemical GUS staining

Fluorometric GUS enzymatic assay to measuring GUS activity in tobacco protoplast extracts or plant tissues, and histochemical GUS analysis in plants were performed following the published protocol [50] as described earlier [51]. The total protein content in plant extracts was assayed with a Bio-Rad protein assay reagent (http://www.bio-rad.com) using BSA as a standard.

### GFP detection

The GFP fluorescence images of electroporated tobacco protoplasts were taken with a fluorescence microscope, and different tissue samples of both transgenic *Arabidopsis* and tobacco plants expressing promoter-reporter gene constructs were analyzed with a confocal laser scanning microscope (TCS SP5; Leica Microsystems CMS GmbH, D-68165 Mannheim, Germany) using LAS AF (Leica Application Suite Advanced Fluorescence) 1.8.1 build 1390 software under PL FLUOTAR objective (10.0X/N.A.0.3 DRY) with confocal pinhole set at Airy 1 and 1× zoom factor for improved resolution with eight bits as described earlier [45]. Excitation of the expressed GFP in transgenic plants was with an argon laser (30%) with AOTF for 488 nm (at 40%) [52] and fluorescence emissions collected between 501 and 580 nm detector gain set at 1050 V.

### SDS-polyacrylamide gel electrophoresis and Immunoblot analysis

SDS-polyacrylamide gel electrophoresis was performed using 10% polyacrylamide gels according to an earlier literature [53]. Forty µg of protein from leaf, stem, flower and root tissues from transgenic tobacco plants were subjected to SDS 10% polyacrylamide gel electrophoresis for western blotting. The Rubisco large subunit (LSU) was stained by Ponceau S stain as an internal control for loading uniformity.

For determination of GFP expression in different tissues, western blot analysis was done using anti GFP from Santa Cruz Biotechnology (Santa Cruz, CA) and horseradish peroxidase-conjugated anti-rabbit secondary antibody (1∶5000) and detected using a chemiluminescent reagent (Pierce Biotechnology, Rockford, IL) following a published protocol [46].

### Conditions for stress treatments

For qRT-PCR analysis of *At4g35985* and *At4g35987* transcripts, three-week-old *Arabidopsis* seedlings grown on ½ MS plates were transferred to liquid ½ MS medium for various stress treatments for 6 hr and 24 hr following an earlier protocol [54]. Whole plants were treated with stress agents: salt stress (150 mM and 200 mM NaCl), osmotic stress (300 mM and 500 mM Mannitol), or cold (4°C).

For analyzing the expression of *Arabidopsis* promoter-reporter constructs, three-week-old *Arabidopsis* seedlings grown on ½ MS plates were transplanted on 150 and 200 mM NaCl plates and allowed to grow an additional 3 d and subsequently analyzed for GUS enzymatic activity, transcript abundance, and histochemical staining. For histochemical GUS staining of flower and stem tissues, *Arabidopsis* seedlings at the flowering stage were irrigated with 200 mM NaCl for 3 d and subsequently analyzed.

For analyzing salt stress effect in tobacco promoter-reporter construct bearing plants, four-week-old tobacco seedlings cultured on rooting media (T- media) were transferred to media supplemented with 150 mM NaCl. After 5 d of NaCl exposure, samples were collected for analysis.

### Determination of transcription start site of the bidirectional promoter (P85–P87) by 5′-RACE analysis

The transcription start site of the bidirectional promoter (P85–P87) was determined by 5′-rapid amplification of cDNA ends (5′-RACE). Total RNA was isolated from transgenic tobacco seedling for the construct pKGFP::P85–P87::GUS. 5′-RACE for the P87 promoter was carried out using GUS specific reverse primer (5′-d-CGCGTGGTTACAGTCTTGCGCGACA-3′) for first strand cDNA synthesis followed by two nested reverse primers (5′-d-CACAAACGGTGATACGTACACTTTT-3′ and 5′-d-TTTCGCGATCCAGACTGAATGCCCACAGGCCGTCGA-3′) in subsequent steps following an earlier protocol [30]. For 5′-RACE analysis of the P85 promoter a GFP specific reverse primer (5′-d-GAAGAAGAACTGGTCCATCTC-3′) was used for first strand cDNA synthesis and subsequently two nested reverse primers (5′-d-TTGAAATCGATGCCCTTAAGC-3′ and 5′-d-AGTCACAAGAGTTGGCCAA GG-3′) from the GFP sequence were used following a protocol published earlier [30].

### Biotic stress treatment

For determining the activation of the bidirectional promoter region under biotic stress conditions, leaves from 2 month old tobacco plants bearing the GFP::P85–P87::GUS construct were used. Spores from *Peronospora tabacina* isolate KY79 were collected and the concentration adjusted to 5000 spores/ml as described earlier [26]. Two to three leaves per plant were inoculated with drops of the spore suspension (containing 50 or 100 spores per inoculum) as described earlier [26]. The leaves of transgenic plants taken as mock or without infection control for the biotic stress treatment were inoculated with an equal amount of water [26]. The promoter activity was analyzed after 24 h of inoculation by histochemical GUS staining as well as by analyzing GUS enzymatic activity in the inoculated leaves following an earlier protocol [55].

## Results

### Structure and sequence analysis of the bidirectional promoter

In *Arabidopsis* chromosome 4, an intergenic region (IR, 1258 bp, genomic coordinates 17033863 to 17035120) shared by two adjacent divergently oriented genes (*At4g35985*, senescence associated gene; and *At4g35987*, calmodulin methyl transferase gene), is a putative bidirectional promoter. *In silico* (bioinformatics) analysis of the 1258 bp putative bidirectional promoter revealed the presence of the following *cis*-regulatory elements detected by PLANTCARE and PLACE listed in Table 1. One stress related element (as-1 element, TGACG), one methyl jasmonate responsive element (CGTCA), nine salicylic acid responsive elements (one W-box and eight W-box core elements), nine Dehydration or senescence associated *cis*-elements including one ABRE like sequence and eight as-2 elements, one drought responsive elements (MBS; TAACTG) and four GT-1 boxes (GAAAAA), responsible for NaCl or pathogen responsiveness were identified in the promoter. A number of elements responsible for tissue specific expression like 8 root specific elements (ATATT), thirteen elements for pollen specific expression (AGAAA) and nine GTGA-motifs responsible for late pollen development and pectate lyase activity were identified in the promoter module. Additionally 9 light responsive elements including Box I, G-box, GATA-motif, GT1-motif, TCT-motif, SORLIP1AT and SORLIP2AT were detected in the promoter region. The bidirectional promoter was found to possess a TATA-box (TATAAA) in the negative strand at 69 to 64 bp upstream from the start codon of *At4g35985* gene and a CAAT-box (CAAT) in the sense strand at 1088 to 1091 bp from the start codon of *At4g35985* gene. Interestingly the CAAT-box was 167 bp upstream from the start codon of the *At4g35987* gene.

**Table 1:** List of various *cis*-regulatory elements and their positions in theP85–P87 bidirectional promoter.

1	Defense and stress	ASF-1 or as-1	TGACG	Cauliflower mosaic virus (Benfey and Chua, 1990)	(1185–1181)	−
2	Methyl jasmonate responsiveness	CGTCA-motif	CGTCA	*Picea glauca* (Germain et al., 2011)	(1181–1185)	+
3	Salicylic acid responsiveness	W-box	TTGACC	*Arabidopsis thaliana *(Eulgem et al., 2000)	(1113–1118)	+
		W-box (core)	TTGAC	*Arabidopsis thaliana* (Yu et al., 2001)	(227–231), (422–426), (447–451), (939–943), (953–957), (1084–1088), (1113–1117)	+
					(798–794)	−
4	Dehydration and senescence	ABRE-like sequence	ACGTG	*Arabidopsis thaliana* (Simpson et al., 2003)	(191–195)	+
		ASF-2 or as-2	GATA	Cauliflower mosaic virus and Arabidopsis (Lam and Chua, 1989)	(182–185), (430–433), (629–632)	+
					(4–1), (293–290), (316–313), (745–742), (936–933)	−
5	Drought responsiveness	MBS	TAACTG	*Arabidopsis thaliana* (Urao et al., 1993)	(147–152)	+
6	NaCl or Pathogen responsiveness	GT-1 box	GAAAAA	*Glycine max* and *Arabidopsis thaliana* (Park et al., 2004)	(77–82), (100–105)	+
					(528–523), (598–593)	−
7	Root specific expression	Root motif	ATATT	*Agrobacterium rhizogenes* (Elmayan and Tepfer, 1995)	(297–301), (340–344), (683–687), (1001–1005)	+
					(300–296), (664–660), (686–682), (1004–1000)	−
8	Pollen specific expression	POLLEN1LELAT52	AGAAA	*Lycopersicon esculentum* (Bate and Twell, 1998)	(93–97), (99–103), (126–130), (533–537), (720–724), (855–859), (1216–1220), (1237–1241)	+
					(326–322), (529–525), (599–595), (706–702), (919–915)	−
9	Late pollen development and pectate lyase	GTGA-motif	GTGA	*Nicotiana tabacum* (Rogers et al., 2001)	(9–12), (118–121), (901–904), (1048–1051)	+
					(384–381), (694–691), (747–744), (995–992), (1102–1099)	−
10	Light	Box I	TTTCAAA	Poplar (Wang et al., 2012)	(80–74), (771–765)	−
		G-box	CACGTC	*Zea mays* (Smykowski et al., 2010)	(195–190)	−
		GATA-motif	GATAGGA	Poplar (Wang et al., 2012)	(182–188)	+
		GT1-motif	GGTTAA	*Pisum sativum* (Green et al., 1988)	(253–248)	−
		TCT-motif	TCTTAC	Poplar (Wang et al., 2012)	(489–494)	+
					(851–846)	−
		SORLIP1AT	GCCAC	*Arabidopsis thaliana* (Hudson and Quail, 2003)	(365–369)	+
		SORLIP2AT	GGGCC		(240–244)	+
11	Binding with RNA transcription factor	CAAT-box	CAAT		(1088–1091)	+
		TATA-box	TATAAA		(69–64)	−

The immediate upstream nucleotide of the start codon of *At4g35985* is designated as position 1 and the immediate upstream nucleotide of the start codon of *At4g35987* is designated as position 1258.

### Transient expression analysis (tobacco protoplast assay and *Agro*-infiltration in *N. Benthamiana* leaf) of the bidirectional promoter P85–P87

**Figure 1:**
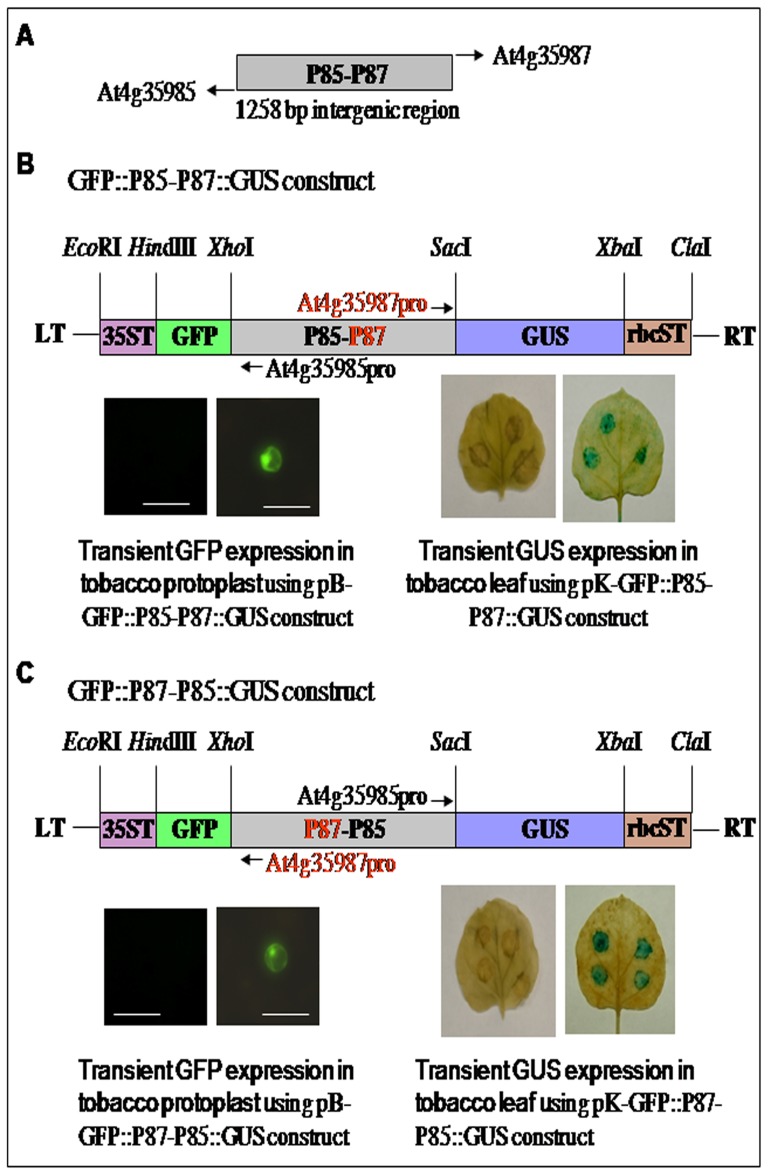
A schematic presentation of the bidirectional promoter (P85–P87), promoter-reporter constructs and transient expression analysis using two reporter genes (GFP and GUS) in both orientations. (A) A schematic map of the bidirectional promoter (P85–P87) located in the intergenic region (IR, 1285 bp, genomic coordinates 17,033,863–17,035,120; TAIR 10) in Arabidopsis chromosome 4, directs two divergent *At4g35985* and *At4g35987* genes arranged in head-to-head orientation. (B–C) Schematic diagram of bidirectional promoter-reporter gene constructs: GFP::P85–P87::GUS and GFP::P87–P85::GUS for transient assay in tobacco protoplasts using pB-GFP::P85-P87::GUS and pB-GFP::P87–P85::GUS and for transient Agro-infiltration experiments using pK-GFP::P85–P87::GUS (GeneBank Accession no. KF661330) and pK-GFP::P87–P85::GUS. Below each construct a representative assay of transient GFP expression detected in fluorescence imaging of tobacco protoplast (bar represents 100 µm) and transient GUS expression detected histochemically by *Agrobacterium* infiltration assay in *N. benthamiana* leaf are shown for respective promoter *At4g35985* promoter (P85) and *At4g35985*7 promoter (P87) activities. Genetic elements: left and right T-DNA border (LT and RT, respectively), CaMV 35S terminator (35ST), green fluorescence protein gene (GFP), β-glucuronidase (GUS), 3′-terminator sequences of ribulose bisphosphate carboxylase small subunit (rbcST) and restriction enzymes *Eco*RI, *Hin*dIII, *Xho*I, *Sac*I, *Xba*I, *Cla*I used to assemble these expression constructs are shown.

The promoter-function of the intergenic region shared by the divergent genes *At4g35985* and *At4g35987* was performed using two reporter genes (GFP and GUS) in both orientations. We designated the *At4g35985* and *At4g35987* promoters as P85 and P87, respectively. As depicted in Figure 1, promoter-reporter gene constructs: pB-GFP::P85–P87-GUS, and pB-GFP::P87–P85::GUS were used for transient tobacco protoplast assays; and constructs pK-GFP::P85–P87-GUS (GenBank Accession no. KF661330) and pK-GFP::P87–P85::GUS were used for transient *Agro*-infiltration assays. In transient tobacco protoplast assays, P87 and P85 gave GUS enzymatic activity of 212±32 pmole and 740±46 pmole of MU/min/mg protein, respectively. Similarly, transient assay in protoplasts with the GFP reporter gene, P85 showed stronger GFP expression (data not shown) as well as GFP fluorescence compared to P87 (Figure 1). In *Agro*-infiltration assays, after two days of post infiltration, histochemical GUS expression was detected in *N. Benthamiana* leaves. The intensity of GUS staining was stronger for the P85 compared to P87, (Figure 1). These results suggest that the 1258 bp intergenic region functions as a bidirectional promoter in a gene independent manner, and that the P85 promoter is relatively stronger than the P87 promoter. The bidirectional promoter activity of the 1258 bp intergenic region was again confirmed by P85 and P87 directed GFP and GUS transient expressions in onion epidermal cells using gene gun transformation (for gene gun experimental procedures see Methods S1; Figure S1).

### Relative expression (transcript) of *At4g35985* and *At4g35987* genes in various *Arabidopsis* tissues

The relative transcript abundance of these two (senescence associated, *At4g35985*; and calmodulin methyltransferase, *At4g35987*) divergent genes were assayed by quantitative real-time PCR (qRT-PCR) using gene specific primers in various tissues (young leaf, older leaf, stem, flower, root and immature silique tissues) to evaluate their possible functional role during development and growth.

**Figure 2:**
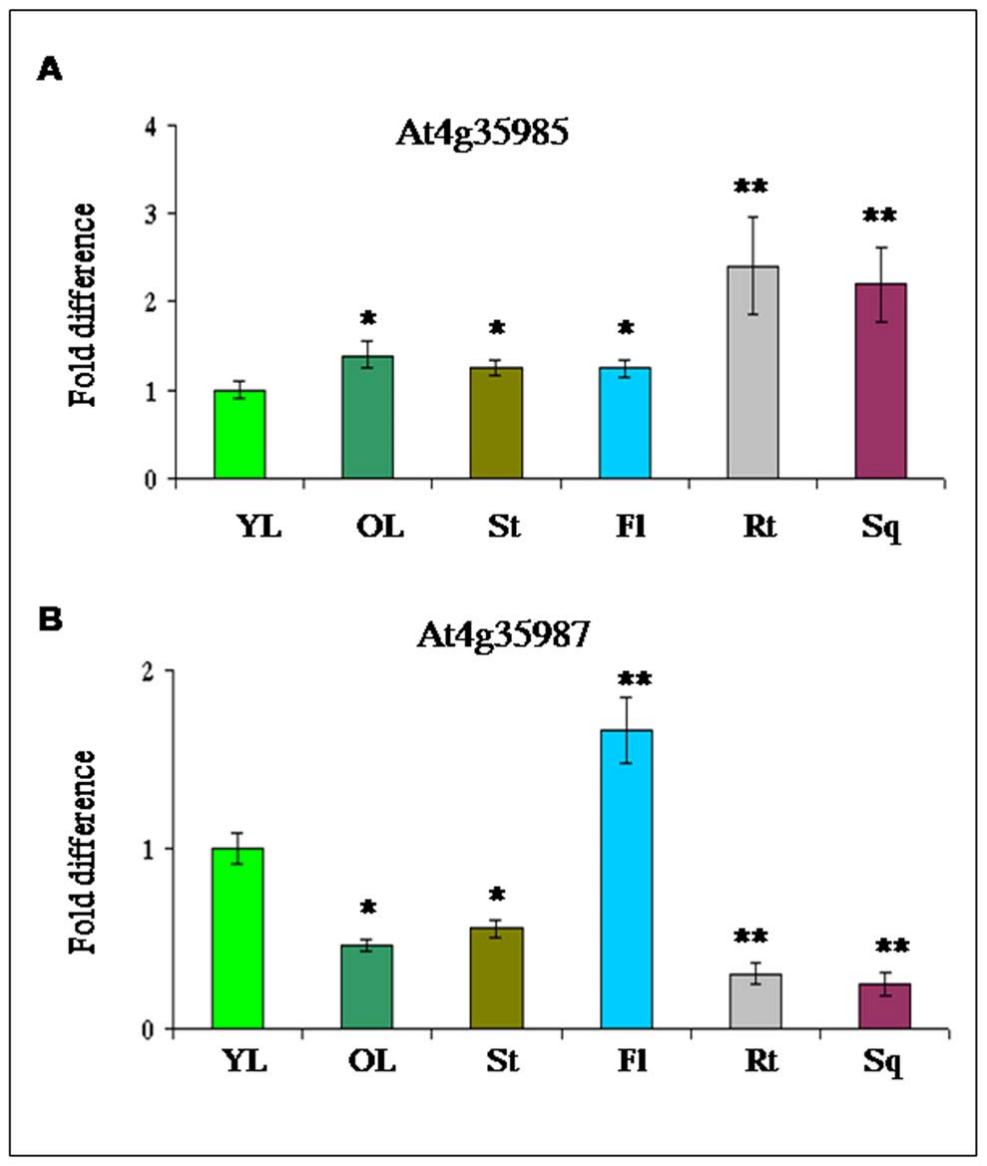
Expression profiling (relative transcript) of *Arabidopsis At4g35985* and *At4g35987* genes in vegetative and reproductive tissues. (A–B) Relative transcript abundance of two *Arabidopsis thaliana* genes, *At4g35985* (A) and *At4g35987* (B) detected in young leaf (YL), older leaf (OL), stem (St), flower (Fl), root (Rt) and immature silique (Sq) tissue by qRT-PCR. Data represent average fold difference of transcript ± SD of three independent experiments with four replicates. Asterisk and double asterisks indicate the significant deviation from young leaf (YL) at P<0.05 and P<0.01, respectively using Student's *t* test for comparison between YL and other tissues separately for both the genes.

The level of expression of the senescence associated *At4g35985* gene was in the following order, maximum in root, followed by immature silique tissue > older leaf > stem > flower tissue > least in young leaf (Figure 2A). Compared to the relative expression of *At4g35985* in young leaves, the relative transcript abundance was significantly higher (P<0.01) in root and immature silique tissues. The *At4g35987* transcript abundance was highest in floral tissues followed by expression in young leaves > stem and older leaves, whereas; the least expression was detected in root and developing silique tissues (Figure 2B). Compared to the relative expression of *At4g35987* in young leaves, the relative transcript abundance was significantly lower (P<0.01) in root and immature silique tissues, and moderately lower (P<0.05) in older leaves whereas significantly higher in the floral tissues (P<0.01). Data indicate that this bidirectional promoter directs gene expression in an orientation dependent manner during *Arabidopsis* development and growth.

### Expression studies of the *At4g35985* and *At4g35987* genes in response to various abiotic stresses

**Figure 3:**
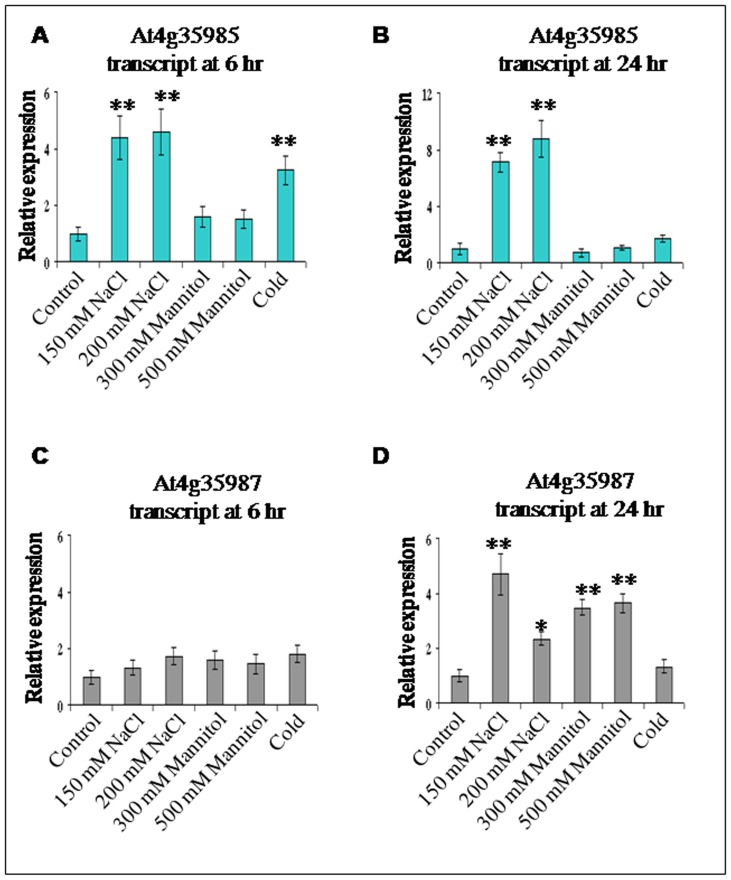
Effect of several abiotic stresses (NaCl salt, mannitol and cold) on expression (transcript) of *Arabidopsis At4g35985* and *At4g35987* genes. (A–D) Expression of the *At4g35985* gene (A–B) and *At4g35987* gene (C–D) under stress treatments. Three-week-old Arabidopsis seedlings (Col-0) were subjected to 150 mM NaCl, 200 mM NaCl, 300 mM Mannitol, 500 mM Mannitol and cold treatment (4°C); after 6 hr and 24 hr of stress treatment, total RNA was extracted from seedling and relative transcript abundance measured by qRT-PCR. Data represents mean ± SD of three biological samples each time with four replicates for various treatments. Asterisk and double asterisks indicate the significant deviation from untreated line (control) at P<0.05 and P<0.01, respectively using Student's *t* test for comparison between control and each treatment separately in all the cases.

Transcript abundance was measured for divergent *At4g35985* and *At4g35987* genes to understand the regulation of expression of these two tissue specific genes under different stress conditions (Figure 3). Salt stress (NaCl) was found to up-regulate the expression of *At4g35985* as early as 6 h and remained effective even after 24 h of treatment (Figure 3A–B). After 6 h of NaCl treatment (150 mM and 200 mM) the transcript of *At4g35985* was up-regulated almost 4 to 4.5 fold (P<0.01) compared to the untreated condition; after 24 h of treatment the expression was almost 6.5 fold and 9 fold in response to 150 mM NaCl and 200 mM NaCl, respectively. Osmotic stress with mannitol (300 mM and 500 mM) for 6 h and 24 h, showed no appreciable changes in the relative abundance of *At4g35985*-specific transcript compared to untreated seedlings. After treatment with cold stress for 6 h the expression of the senescence associated *At4g35985* gene was elevated up to 3 fold (P<0.01) but when the cold stress was extended for 24 h no significant expressional changes were observed compared to untreated samples (Figure 3A–B).

The relative expression of the *At4g35987-*specific transcript did not show any significant changes after 6 h of salt (NaCl), mannitol or cold treatments (Figure 3C). Interestingly, after 24 h of 150 mM NaCl treatment, the *At4g35987* expression was significantly up-regulated (P<0.01) whereas at 200 mM NaCl stress the transcript level was moderately up-regulated (P<0.05), indicating higher salt concentration is less-responsive (Figure 3C–D). After 24 h response to osmotic stress with 300 mM and 500 mM mannitol, the expression of *At4g35987* gene was up regulated significantly (P<0.01) compared to the untreated condition whereas cold treatment did not show any significant changes in the transcript abundance (Figure 3D). These results indicate that two divergent (senescence associated, *At4g35985*; and calmodulin methyltransferase, *At4g35987*) genes are differentially regulated in response to various abiotic stresses as a function of strength of stress and time of exposure.

### Analysis of the bidirectional promoter P85–P87 with reporter genes GUS and GFP in both orientations in transgenic *Arabidopsis* plants

**Figure 4:**
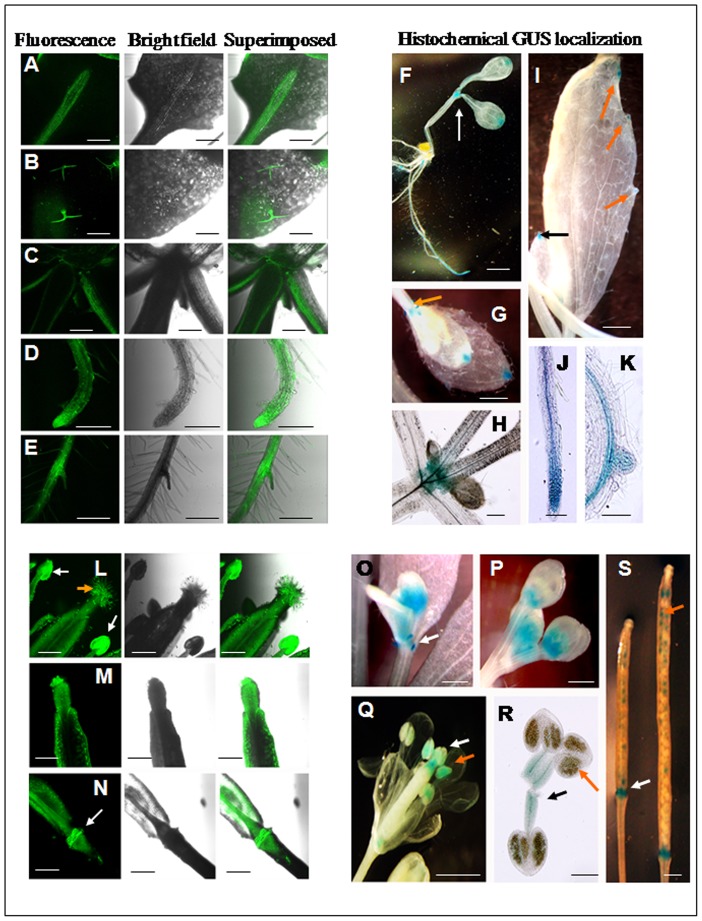
Localization of GFP and GUS in vegetative and reproductive tissues of GFP::P85–P87::GUS transgenic *Arabidopsis* plants. (A–E) Confocal laser scanning microscopic analysis of GFP expression under *At4g35985* promoter (P85) in three-weeks-old *Arabidopsis* seedlings. GFP expression in leaf midrib (A), leaf trichomes (B), apical meristematic region (C), primary root (D) and lateral root (E). Green fluorescence image of GFP (left); bright field image (middle), superimposed image (right) are shown. Bar represents 250 µm in each image. (F–K) Histochemical GUS localization data for the *At4g35987* promoter (P87) in vegetative tissues of *Arabidopsis* plants. (F) GUS expression in the apical meristematic region (indicated by arrow), leaf tip, and root parts in 5-day-old *Arabidopsis* seedlings, bar 1 mm; (G) GUS expression in leaf tip and very young bud (orange arrow) of two-week-old *Arabidopsis* seedlings, bar represents 1 mm; (H) GUS expression at the junction of young bud and stem, bar represents 1 mm; (I) GUS expression in leaf tip (black arrow) and hydathode (orange arrow) at aerial leaves of three-week-old *Arabidopsis* plants; bar represents 1 mm; (J) GUS expression in root tip of five-day-old seedlings, bar represents 100 µm; and (K) GUS expression in root stele and emerging lateral root of five-day-old seedling, bar represents 100 µm, are presented. (L–N) Confocal laser scanning microscopic analysis of GFP expression under the *At4g35985* promoter (P85) in reproductive tissues of *Arabidopsis* plants. GFP expression in anther (white arrow), stigma (orange arrow) in flower (L); tip of immature silique (M); and abscission zone (white arrow) in the base of silique (N) are presented. Green fluorescence image of GFP (left); bright field image (middle), superimposed image (right) are shown. Bar 250 µm in each image. (O–S) Histochemical GUS detection for *At4g35987* promoter (P87) in reproductive tissues of *Arabidopsis* plants. (O) GUS expression in emerging floral bud (indicated by white arrow), bar represents 1 mm; (P) GUS expression in developing floral bud, bar represents 1 mm; (Q) GUS expression in stigma (white arrow) and anther (orange arrow) of *Arabidopsis* flower, bar represents 1 mm; (R) GUS expression in filament (black arrow) and pollen grains in anther (orange arrow), bar represents 200 µm; (S) GUS expression in abscission zone (white arrow) and immature seeds (orange arrow) of silique, bar represents 1 mm.

To evaluate the tissue specific nature of the bidirectional promoter, independent transgenic *Arabidopsis* lines were generated for the constructs pK-GFP::P85–P87::GUS and pK-GFP::P87–P85::GUS. In GFP::P85–P87::GUS plants, among the vegetative tissues strong GFP expression for P85 was detected in midrib of leaves, leaf trichomes, apical meristemic region, throughout the root and lateral roots (Figure 4A–E). On the other hand, strong histochemical GUS staining under P87 was observed in leaf-tip, apical meristem and root-tip regions at cotyledon stage. In addition, the leaf tip, hydathode, bud, apical meristemic region, root tips, emerging lateral root tips and root stele region showed maximum GUS expression as detected by histochemical GUS staining of plants (Figure 4F–K). Similarly, the bidirectional promoter showed expressional differences in the reproductive tissues of Arabidopsis in an orientation dependent manner. In GFP::P85–P87::GUS plants, the GFP expression under P85 showed significant expression in anther, stigma and silique (Figure 4L–N) whereas; GUS expression under P87 was detected in floral bud, stamen, stigma, anther and immature silique (Figure 4O-S).

**Figure 5:**
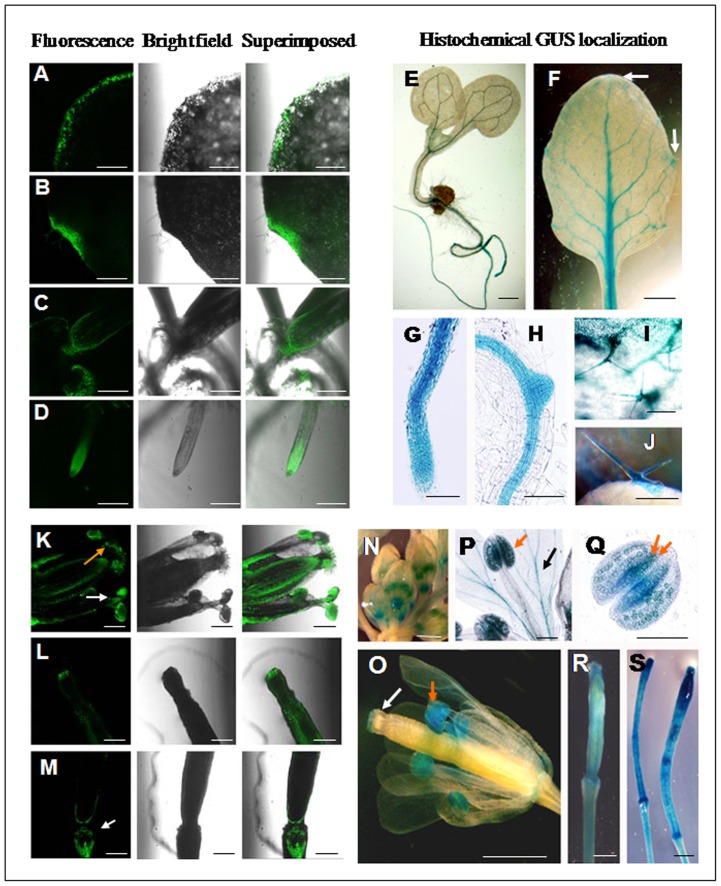
Localization of GFP and GUS in vegetative and reproductive tissues of GFP::P87–P85::GUS transgenic *Arabidopsis* plants. (A–D) Confocal laser scanning microscopic analysis of GFP expression under the *At4g35987* promoter (P87) in *Arabidopsis* plants. GFP expression in peripheral border region of cotyledon in five-days-old *Arabidopsis* seedling (A); hydathode in three-weeks-old seedling (B), apical meristematic region in three-week-old seedlings (C); and primary root tip in five-day-old seedling (D). Green fluorescence image of GFP (left); bright field image (middle), superimposed image (right) are depicted. Bar represents 250 µm in each image. (E–J) Histological GUS localization data for the *At4g35985* promoter (P85) in vegetative tissues of *Arabidopsis* plants. (E) GUS expression in the midrib of cotyledons, young stem and root in five-day-old *Arabidopsis* seedling, bar represents 1 mm; (F) moderate GUS expression in leaf tip, hydathode and strong GUS expression in leaf venation at aerial leaf in three-week-old *Arabidopsis* plants, arrow indicates expression at leaf tip and hydathode, bar represents 1 mm; (G) strong GUS expression in root tip of five-day-old seedlings, bar represents 100 µm; (H) strong GUS expression in root stele and emerging lateral root of five-day-old seedlings, bar represents 100 µm; (I) GUS staining of leaf trichomes. bar represents 200 µm; (J) GUS staining of trichomes in young bud, bar represents 200 µm. (K–M) Confocal laser scanning microscopic analysis of GFP expression under the *At4g35987* promoter (P87) in reproductive tissues of *Arabidopsis* plants. GFP expression in anther (white arrow) and stigma (orange arrow) in flower (K); tip of immature silique (L); and abscission zone (white arrow) in the base of silique (M) are depicted. Green fluorescence image of GFP (left); bright field image (middle), superimposed image (right) are shown. Bar represents 250 µm in each image. (N–S) Histochemical GUS localization under the *At4g35985* promoter (P85) in reproductive tissues of *Arabidopsis* plants. (N) GUS expression in floral bud, bar represents 1 mm; (O) GUS expression in anther (orange arrow) and stigma (white arrow) in *Arabidopsis* flower, bar represents 1 mm; (P) GUS expression in anther (orange arrow) and petals (black arrow), bar represents 200 µm; (Q) GUS expression in pollen grains bearing anther, pollen grains are indicated by orange arrow. bar represents 200 µm; (R) GUS expression in developing silique. bar represents 1 mm; (S) GUS expression in developed silique before senescence, bar represents 1 mm.

Interestingly among the vegetative tissues, transgenic plants developed with the construct pK-GFP::P87–P85::GUS where reporter genes GFP and GUS were expressed under P87 and P85 respectively; GFP expression under P87 was located in the border region of cotyledons and in the mature leaf but most of the expression was detected in hydathode (Figure 5A–B). Additionally GFP expression under P87 was detected in the shoot apical meristemic region and root tip (Figure 5C–D). Whereas P85 showed strong GUS localization in mature leaf veins and hydathode region, relatively less expression in mature leaf tip and cotyledonary leaves, detection of GUS was mostly observed throughout the root, emerging lateral root tip and leaf trichomes (Figure 5E–J).

In reproductive tissues of GFP::P87–P85::GUS plants, the GFP expression under P87 was mostly detected in anther, stigma and base of the silique (Figure 5K–M) whereas; under P85 strong GUS expression was detected in the anther, stigma, pollen grains, developing and immature silique and faint GUS expression was observed in petals (Figure 5N–S).

No detectable GFP fluorescence was visualized in untransformed control Arabidopsis plants in vegetative tissues (Figure S2) whereas, negligible fluorescence (might be due to auto-fluorescence) was observed in floral tissues of control plants (Figure S3).

The tissue specific expression pattern of the bidirectional promoter P85–P87 was confirmed with both reporter genes (GUS and GFP). The P85 is stronger compared to P87 in most of the vegetative tissues except the cotyledons. Remarkably, the GUS as well as GFP expression under P85 was stronger compared to P87 expression in reproductive tissues also. Results of GUS and GFP localization studies indicate the orientation dependent expression of the bidirectional promoter P85–P87 during plant development and growth.

### Comparative expression analysis of the bidirectional promoter P85–P87 with GUS reporter gene in transgenic *Arabidopsis* plants

**Figure 6:**
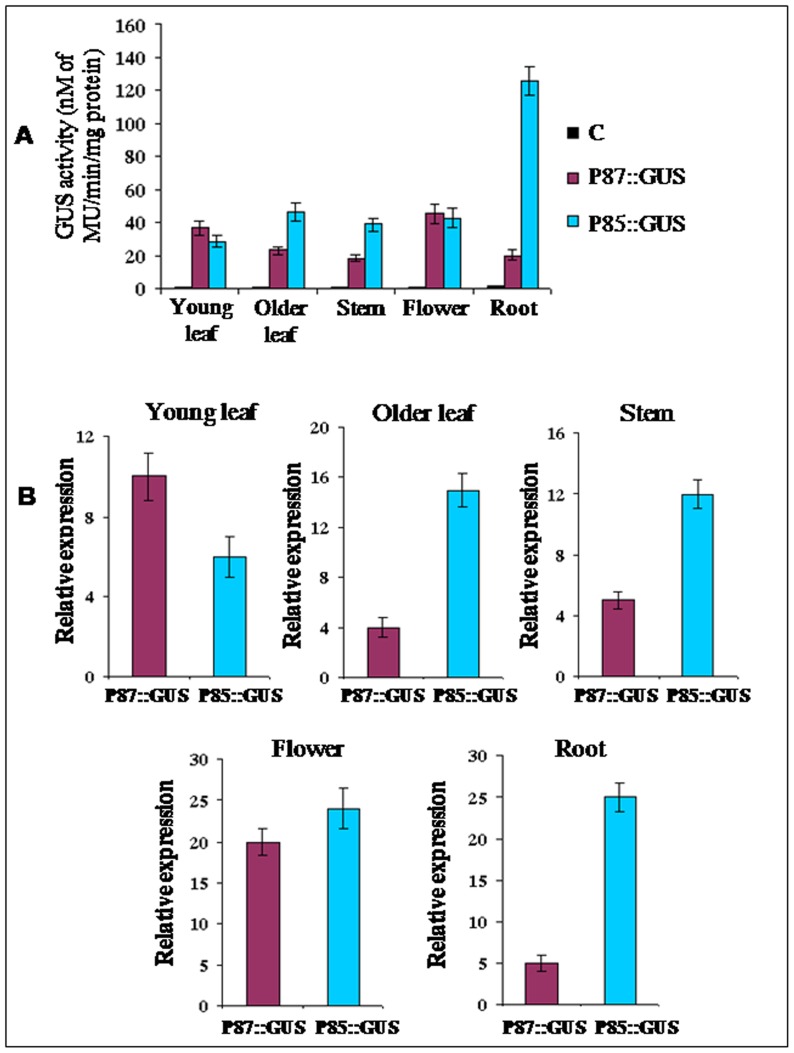
GUS expression in transgenic *Arabidopsis* plants generated for constructs GFP::P85–P87::GUS and GFP::P87–P85::GUS. (A) GUS enzymatic activity of GFP::P85–P87::GUS (represented as P87::GUS) and GFP::P87–P85::GUS (shown as P85::GUS) *Arabidopsis* plants (T_2_ generation) expressing GUS under P87 and P85, respectively was measured in young leaf, older leaf, stem, flower and root tissues. Soluble protein extracts isolated from different plant tissues were used for GUS assay along with the wild type plants (C). Data represents mean ± SD of three biological replicates for each tissue (n = 3). (B) Relative expression of GUS specific transcripts was measured in young leaf, older leaf, stem, flower and root tissues by qRT-PCR in GFP::P85–P87::GUS (represented as P87::GUS) and GFP::P87–P85::GUS (shown as P85::GUS) *Arabidopsis* plants (T_2_ generation) expressing GUS under P87 and P85, respectively. Data represents relative expression of GUS transcript ± SD of three biological replicates for each tissue (n = 3).

To compare bidirectional promoter activity in both directions (P85 and P87), GUS enzymatic activity and GUS-transcript abundance were measured in different tissues of the independent T_2_ generation transgenic *Arabidopsis* lines generated for the constructs pK-GFP::P85–P87::GUS and pK-GFP::P87–P85::GUS at various stages of development and growth (Figure 6). For the P87, assay of GUS enzymatic activity in floral and young leaf tissues showed 45.2±6 and 36.4±4.2 nM of MU/min/mg protein, respectively, and lesser activities in older leaf, stem and root tissues. In contrast, the GUS activity in other orientation driven by the P85 showed maximum activity in root tissue (125.4±8.5 nM of MU/min/mg protein) and least in the young leaf (28.6±3.5 nM of MU/min/mg protein). Both promoters showed almost the same level of GUS activity in floral organs. GUS enzymatic activities in older leaves and stem tissues under P85 were about 2 fold more than the activities driven by the P87 of the bidirectional promoter P85–P87 (Figure 6A).

Relative abundance of GUS specific transcripts were detected by qRT-PCR in independent transgenic *Arabidopsis* lines generated for the constructs pK-GFP::P85-P87::GUS and pKGFP::P87–P85::GUS. Relative abundance of GUS specific transcript under P85 in older leaf, stem and root tissues was 3.8, 2.4, 5 folds higher, respectively, compared to P87. On the contrary, in the young leaf the GUS transcript abundance under P87 was 1.5–2 folds higher than the P85. In floral tissue no significant differences were observed in transcript abundance in both orientations (Figure 6B). Results showed the bidirectional promoter tested with reporter genes directs gene expression in various tissues in a mutually exclusive manner as noted in transcript analysis of the native genes (data in Figure 2).

### The 3′-end deletion analysis of the bidirectional promoter P85–P87::GUS in transient tobacco protoplast system

**Figure 7:**
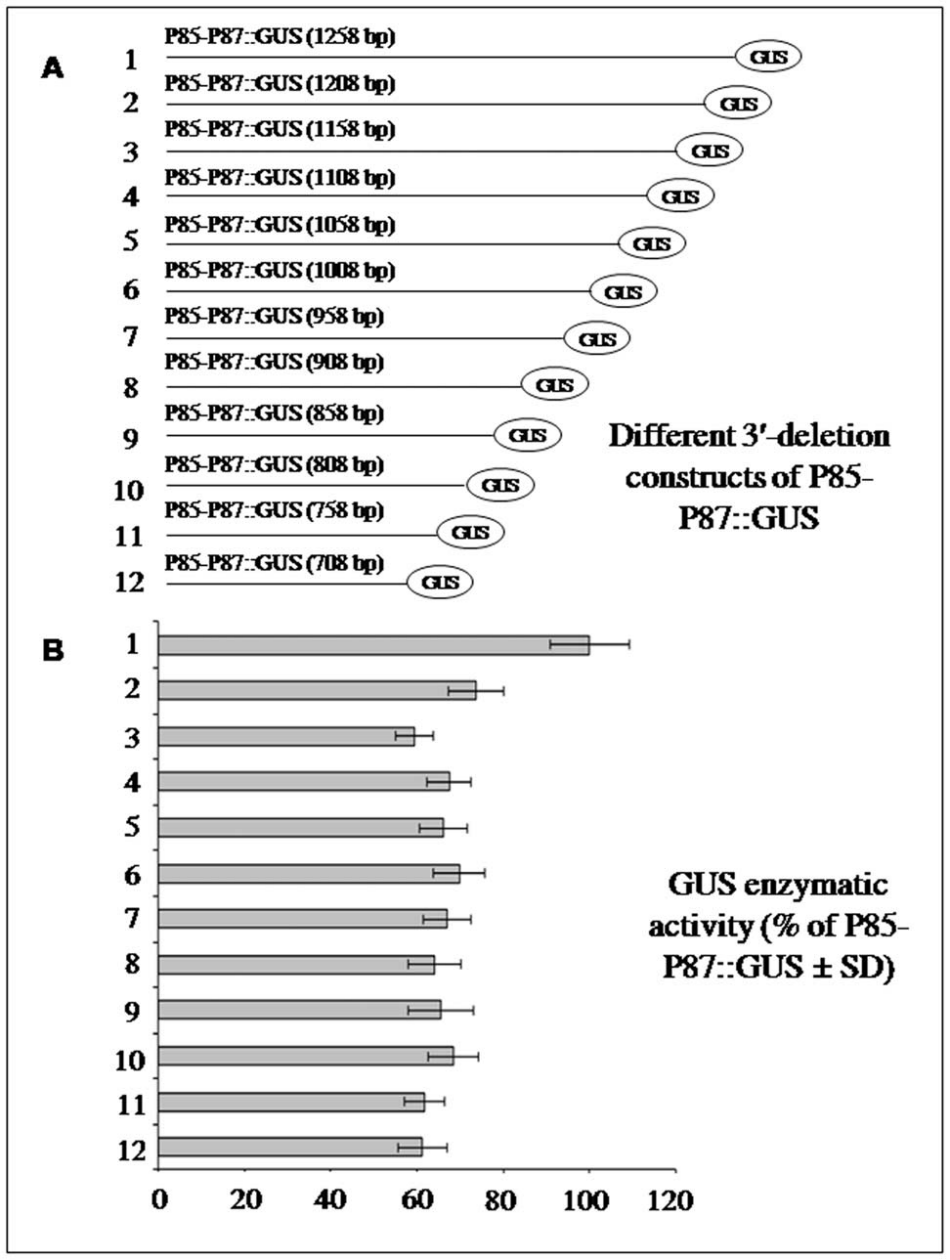
The 3′-end deletion analysis of P85–P87::GUS in transient tobacco protoplast assay. (A) Schematic map of GUS constructs (number 1 to 12) developed for analyzing the regulatory elements present in the 3′-end of 1258 bp (P85–P87) promoter fragment. Construct 1 represents full-length (1258 bp) whereas successive 3′-deletion constructs #2 to 12 with 50 bp less at the 3′-end than the previous one. Constructs #2 to 12 with fragment size 1208, 1158, 1108, 1058, 1008, 958, 908, 858, 808, 758 and 708 bp promoter, respectively, as shown in diagram. (B) Transient GUS expression analysis of constructs 1 to 12 in pKYLX-80 background in tobacco protoplast. The average GUS activity ± SD was presented in the histogram of 3 independent experiments of 3 replications of each construct by taking the activity of full-length (1258 bp) promoter fragment as 100%.

The 3′-end deletion analysis of the P85–P87::GUS depicted that the expression is reduced to ∼60% compared to the whole fragment after deletion of 100 bp from 3′-end (Figure 7). In this region of the *At4g35987* promoter (in P85–P87), a stress responsive and root specific *cis*-sequence (as-1 element, TGACG) is present (Table 1) on the -ve strand that might be responsible for promoter function in stress response and tissue specific expressions.

### Analysis of the bidirectional promoter P85–P87 with reporter genes (GFP and GUS) in transgenic tobacco plants

**Figure 8:**
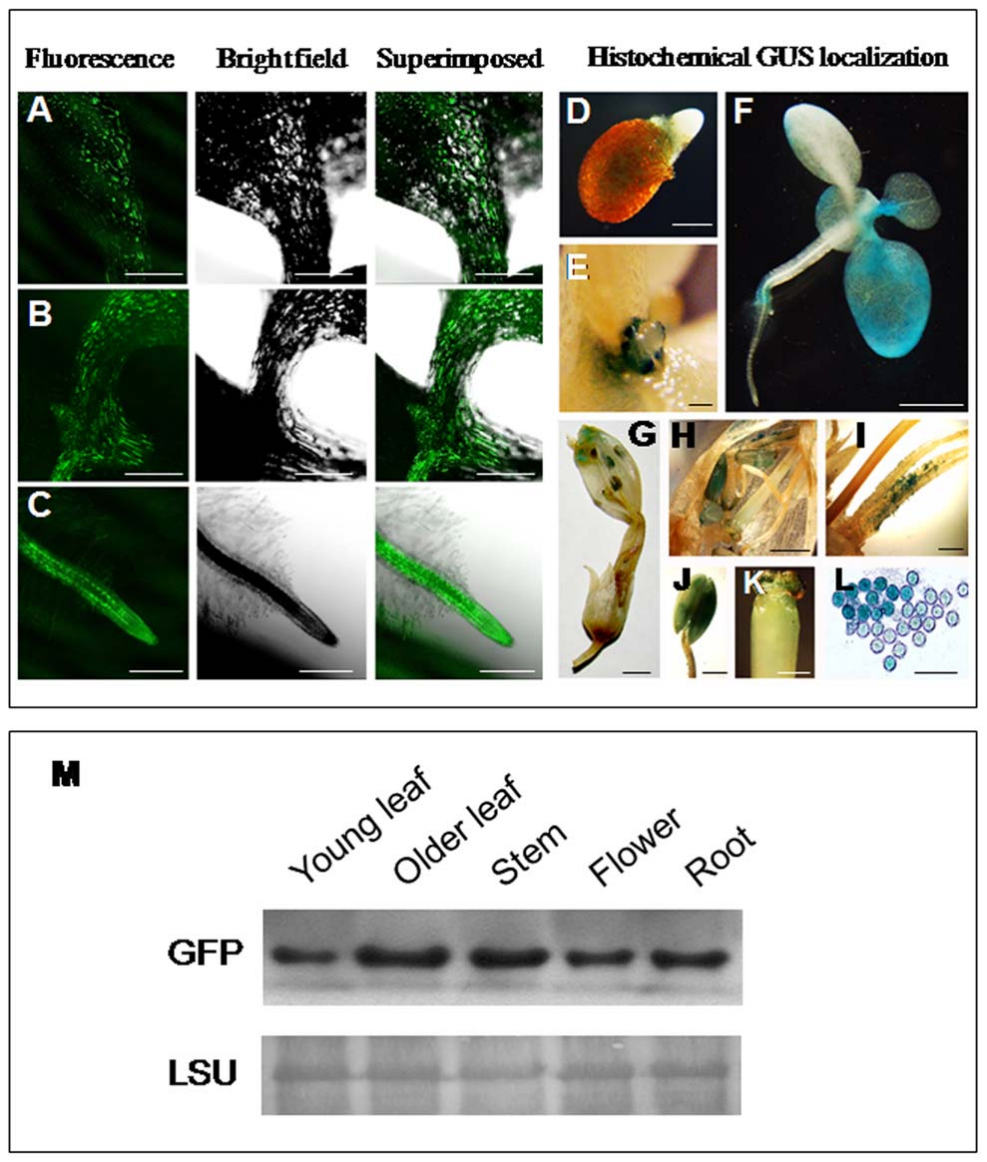
GFP and GUS localization in transgenic tobacco plants generated for the construct GFP::P85–P87::GUS. (A–C) Confocal laser scanning microscopic analysis of GFP expression under the *At4g35985* promoter (P85) in 21-day-old tobacco seedlings (T_2_ progeny). GFP expression in leaf midrib (A), apical meristematic region (B) and primary root (C) are presented. Green fluorescence image of GFP (left); bright field image (middle), superimposed image (right) are shown. Bar represents 250 µm in each image. Histochemical GUS detection under *At4g35987* promoter (P87) in transgenic tobacco plants, GUS expression in germinating tobacco seeds (D) and young primordia at 14-day-old seedlings (E), bar represents 1 mm. (F to H) GUS expression in 21-day-old tobacco seedlings (F); GUS expression in tobacco flower (G); and reproductive tissues of tobacco flower (H); bar represents 5 mm. (I to K) GUS expression in the base of the filament of tobacco flower (I), anther (J) and stigma (K), bar represents 1 mm. (L) GUS expression in pollen grains, bar represents 100 µm. (M) Immunoblot analysis of GFP protein accumulation in various tissue of transgenic tobacco plants generated for the construct GFP::P85–P87::GUS expressing GFP under P85. Western blot showing GFP expression in young leaf, older leaf, stem, flower and root tissues of tobacco plant (T_2_ generation). Forty µg of total protein from different tissues were subjected to 10% polyacrylamide gel electrophoresis, anti GFP antibody from Santa Cruz Biotechnology (Santa Cruz, CA) was used as primary antibody and horseradish peroxidase-conjugated anti-rabbit secondary antibody (1∶5000) in western blot. Lower panel represents the loading control by detecting Rubisco large subunit (LSU) using Ponceau S stain.

To evaluate the functionality and tissue specific nature of the bidirectional promoter in heterologous system, independent transgenic tobacco lines were generated for the constructs pK-GFP::P85–P87::GUS. The GFP expression under P85 was detected in leaf-midrib, apical meristem and root (Figure 8A–C). Empty vector control plants did not show any green fluorescence under similar experimental condition (Figure S4).

During growth and development, the tissue specific expression of GUS under P87 in transgenic tobacco depicted very faint GUS expression in germinating tobacco seeds (Figure 8D) but strong GUS expression was detected in young primordia of tobacco seedlings (Figure 8E). In 3-week-old tobacco seedlings, moderate expression in young leaves and less expression in roots (Figure 8F). In floral tissues of transgenic tobacco plants, GUS activity under P87 was stronger in anther and base of the filament, moderate to strong in pollen grains and lesser in the stigma (Figure 8G–L). Western blot analysis using anti GFP antibody revealed that the GFP expression was strong in older leaves, stem and root tissues and least in young leaves whereas the expression was moderate in floral tissues (Figure 8M).

### Analysis of the bidirectional promoter (P85–P87) fused with reporter genes GUS) in response to salt stress in transgenic *Arabidopsis* and tobacco plants

**Figure 9:**
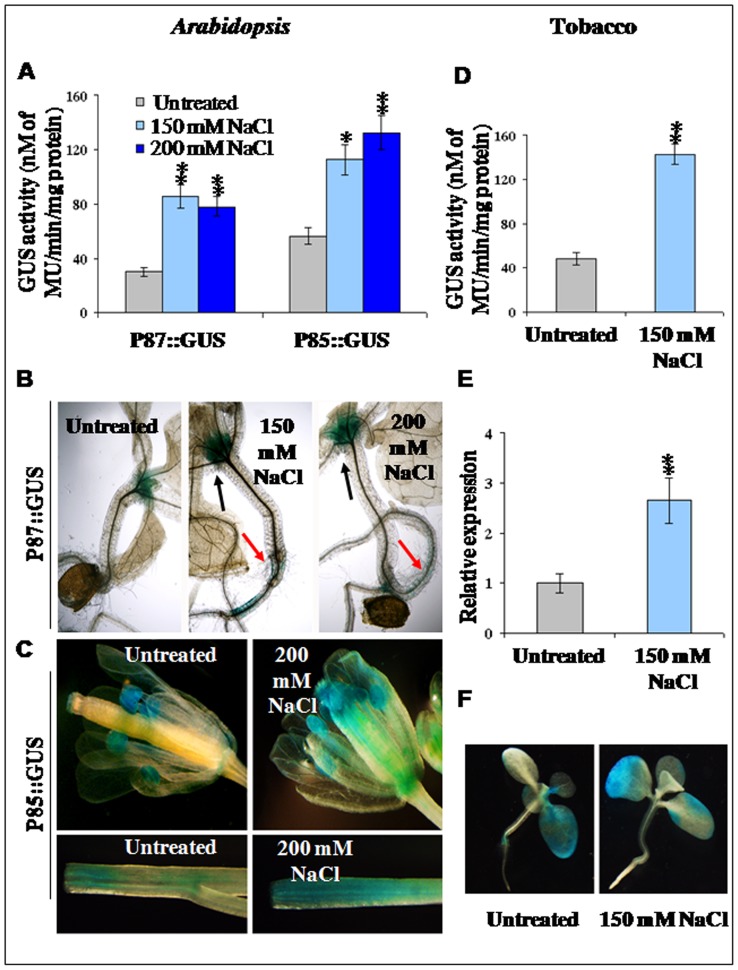
Effect of NaCl stress on GUS expression in transgenic *Arabidopsis* and tobacco plants. (A) Transgenic GFP::P85–P87::GUS and GFP::P87–P85::GUS *Arabidopsis* plants (T_2_ generation) expressing GUS under P87 (P87::GUS) and P85 (P85::GUS), respectively were subjected to 150 mM and 200 mM of NaCl stress for 3 d. Enzymatic GUS activity was measured, asterisk and double asterisks indicate the significant deviation from untreated plants at P<0.05 and P<0.01, respectively using Student's *t* test for comparison between untreated and each treatment separately in all the cases. (B) Histological GUS staining of transgenic *Arabidopsis* GFP::P85–P87::GUS seedlings expressing GUS under P87 (P87::GUS), after 150 mM and 200 mM NaCl stress for 3 d showed strong GUS staining in the apical meristem (black arrow) and root (orange arrow). (C) Histological GUS staining of transgenic *Arabidopsis* GFP::P87–P85::GUS plants expressing GUS under P85 (P85::GUS), after 200 mM NaCl irrigation for 3 d showed elevated GUS localization in floral tissues (upper panel) and stem (lower panel). (D) Effect of 150 mM of NaCl exposure for 5 d on GUS expression in transgenic tobacco GFP::P85–P87::GUS lines (4-week-old seedlings, T_2_ generation) expressing GUS under P87. Enzymatic GUS activity was measured, double asterisks indicate the significant deviation from untreated plants at P<0.01 using Student's *t* test for comparison between untreated and treated one. (E) The relative abundance of GUS transcript detected by qRT-PCR in transgenic tobacco GFP::P85–P87::GUS lines (4-week-old seedlings, T_2_ generation) expressing GUS under P87 after 150 mM NaCl stress for 5 d. Data represent average fold difference of GUS transcript ± SD of three independent experiments with four replications. Double asterisks indicate the significant deviation from untreated plants at P<0.01 using Student's *t* test for comparison between untreated and treated one. (F) Histological GUS staining of tobacco plants after treatment of 150 mM NaCl for 5 d along with the untreated seedling.

Transcript analysis showed that the adjacent divergent genes (*At4g35985* and *At4g35987*) in *Arabidopsis*, are responsive to various abiotic stresses (Figure 3). The abiotic stress responsive nature of the bidirectional promoter fused with reporter genes was evaluated in transgenic *Arabidopsis* and tobacco plants. Independent transgenic *Arabidopsis* and tobacco plants were generated for the construct pK-GFP::P85–P87::GUS and pK-GFP::P87–P85::GUS and GUS activities under P87 and P85 promoters were detected in *Arabidopsis* and tobacco lines after exposing the transgenic plants to NaCl stress (Figure 9).

Analysis of salt (150 mM and 200 mM NaCl,) stress in transgenic *Arabidopsis* lines (3-week-old seedlings, T_2_ generation) exposed for 3 days showed about 2.5 to 3 fold more GUS activity compared to untreated plant for both P87 and P85 promoters (Fig 9A). Comparative GUS-expression data showed that the P85 promoter is stronger than P87 promoter, and at 200 mM NaCl the P85 activity due to salt stress is about 2 fold more compared to P87 (Fig. 9A). Results suggest that the regulatory region in the bidirectional promoter P85–P87 is responsive to abiotic stress in both orientations.

For finer analysis, the effect of salt stress on the growth and development of plants and tissue specific expression of the bidirectional P85–P87 promoter was evaluated in transgenic *Arabidopsis* plants by histochemical GUS staining (Figure 9B–C).

For P87 in GFP::P85–P87::GUS *Arabidopsis* plants (3-week-old, T_2_ generation), after exposure to 150 mM and 200 mM of NaCl for 3 d, stronger GUS expression was observed in the apical meristematic region (between the two upper leaves) and in root tissues compared to the untreated plants (Figure 9B). Whereas at the early stages of growth and development, the functional activity of P85 in GFP::P87–P85::GUS *Arabidopsis* plants (3-week-old, T2 generation) exposed to NaCl treatment showed no distinguishable tissue specific expressional changes (data not shown). Interestingly, full-grown plants at the flowering stage exposed to 3 d of salt stress showed up-regulated tissue specific GUS expression in stem and floral tissues. The GUS expression in different floral parts (anther, stigma, petal and filament) and stem tissues were stronger after salt treatment compared to untreated plants (Figure 9C) as detected by histochemical GUS staining.

In the transgenic GFP::P85–P87::GUS tobacco (4-week-old, T_2_ generation) plants, the GUS enzymatic activity after 150 mM NaCl stress for 5 d was about 3 fold more compared to untreated plants (Figure 9D). The abundance of GUS transcripts under P87 in transgenic GFP::P85–P87::GUS tobacco plants exposed to 150 mM NaCl for 5 d, was elevated around 2.5 to 3 fold compared to untreated plants (Figure 9E). Histochemical GUS staining of transgenic tobacco seedlings exposed to 150 mM NaCl stress also showed more GUS expression (Figure 9F) compared to the untreated one. Results suggest the tissue specific and stress responsive regulatory elements of the *Arabidopsis* bidirectional promoter P85–P87 are functionally active in the heterologous tobacco system.

### Transcription start site (TSS) analyses from both orientations of the bidirectional promoter

The transcription start site of the bidirectional promoter (P85–P87) was determined by 5′-rapid amplification of cDNA ends (5′-RACE) as described in methods. The transcript start site (TSS) for P87 was mapped to a guanine (G) residue located 93 bp upstream from the start codon (ATG) of *At4g35987* gene, and 74 bp downstream from CAAT box. The major TSS for P85 (opposite orientation of P87 in the bidirectional promoter) was mapped to a guanine (G) residue located 33 bp upstream from the start codon (ATG) and 30 bp downstream of the TATA element (TATAAA) of the *At4g35985* gene (Figure S5). In addition to that a number of TSS was detected for *At4g35985* gene (data not shown).

### Biotic stress inducible expression of P85–P87 promoter

**Figure 10:**
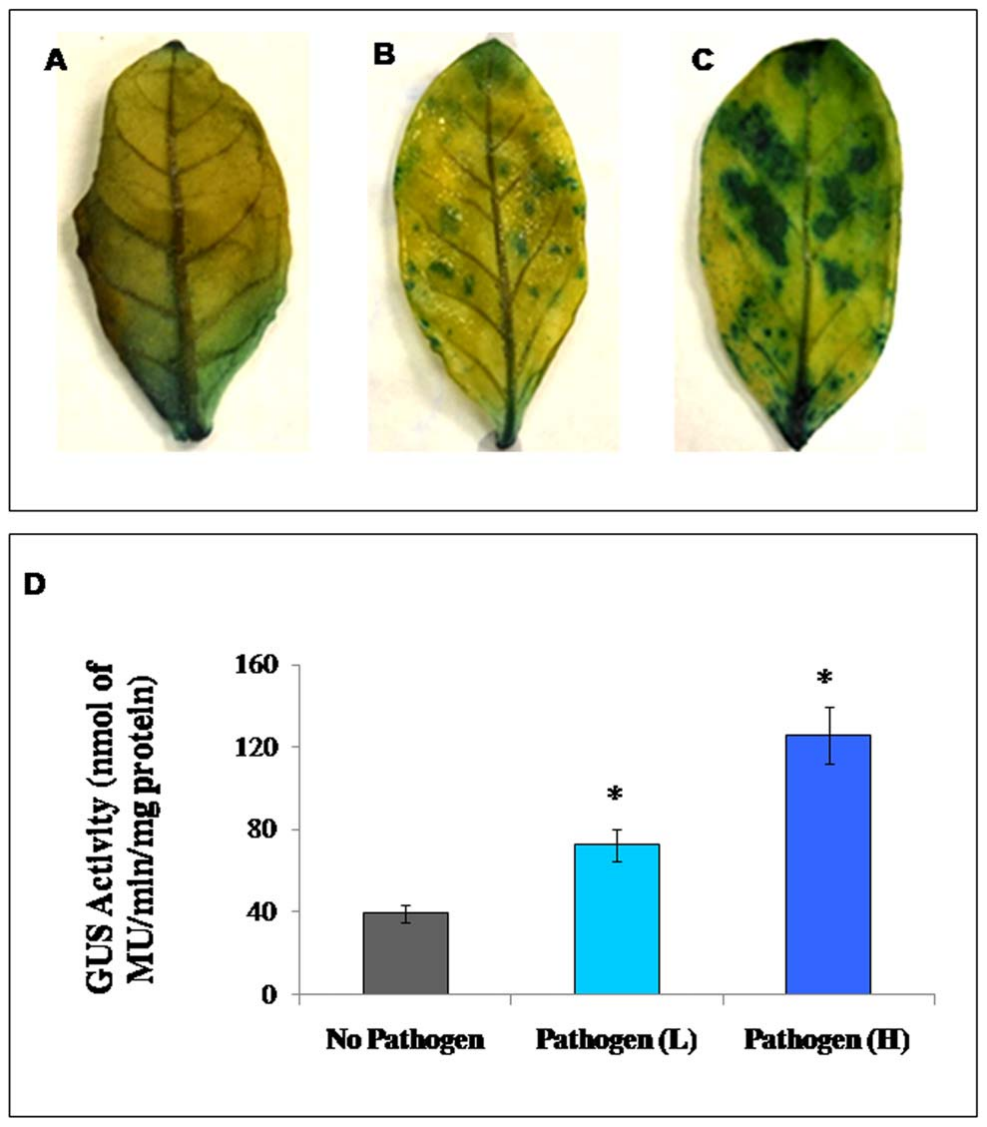
Effect of biotic stress on GUS expression in transgenic tobacco plants. (A–C) To demonstrate the local induction of bidirectional promoter (P85–P87), leaves of T_2_ generation transgenic tobacco plant bearing GFP::P85–P87::GUS construct were inoculated with mock/water (A) along with lower (B) and higher (C) concentrations of tobacco blue mold spores. The promoter activity was monitored after 24 h of inoculation by histological GUS staining. (D) Effect of biotic stress (by blue mold) on GUS expression in leaves of transgenic tobacco GFP::P85–P87::GUS plants. Enzymatic GUS activity was measured, asterisks indicate the significant deviation from water treated plants at P<0.01 using Student's *t* test for comparison between water treated and treated one. No Pathogen: Plant leaves treated with water; Pathogen (L): Plant leaves inoculated with lower concentrations of blue mold spores; Pathogen (H): Plant leaves inoculated with higher concentrations of blue mold spores.

Inoculation of *P. tabacina* was found to induce the P85–P87 promoter activity as revealed by the GUS histochemical assay of transgenic GFP::P85–P87::GUS tobacco plant leaves. Compared to the uninfected tobacco leaves the GUS expression was elevated in the pathogen inoculated leaves in a dose dependent manner (Figure 10). GUS enzymatic assay also confirmed that due to biotic stress induction by *P. tabacina* the promoter activity has been increased from 1.8 to 3.2 fold compared to the mock or uninfected leaves (Figure 10D).

## Discussion

In recent years the relevance of non-coding DNA in phenotypic evolution has been documented [56]. The orientation of flanking genes may influence the evolution of intergenic regions in which *cis*-regulatory elements are likely to be located [57]. Head-to-head clustering of genes where two adjacent genes are separated by a short intergenic distance, and oriented in divergent transcriptional configuration, are prevalent and conserved in many eukaryotes, including yeasts, plants, invertebrates, and vertebrates [58]. Bidirectional gene pairs account for a large proportion (13.3%) of all the *Arabidopsis thaliana* genes, confirming that this structure is also prevalent in plant genomes [5]. The most likely proposed function of such clustering is to co-regulate two adjacent genes by a single bidirectional promoter. A number of bidirectional promoters have been isolated from *Arabidopsis* as well as other plant systems [8], [15]–[17]. The intergenic region (IR) between At5g06290 and At5g06280 directs expression in different spatially-localized tissues of *Arabidopsis* in a mutually exclusive manner [17] whereas; the IR between the cab1 and cab2 genes acting as the bidirectional promoter is equally active in both directions [8]. Another group of researchers demonstrated the orientation dependent expression of oleosin promoter where one side of the promoter was induced by abscisic acid (ABA) while the other side was induced by ethylene [12]. In the present study, the functional activities of an *Arabidopsis* bidirectional promoter (P85–P87, 1258 bp) sharing an intergenic region (IR) between the senescence associated gene (*At4g35985*) and calmodulin methyl transferase gene (CaM KMT; *At4g35987*) at chromosome 4 were critically analyzed.

Structural analysis of the bidirectional promoter depicts that the promoter is governed by CAAT box in one direction (P87) and TATA box in other orientation (P85) (Figure S5). In P87 the TSS (transcription start site) exactly matches the annotation done by TAIR (http://www.Arabidopsis.org/). In P85 a number of TSS was detected and that might produce pervasive transcript or cryptic unstable transcript (CUT), which is yet to be established. Recently a number of studies reported the occurrence of pervasive transcript or CUTs arising from bidirectional promoters [6], [9].

The localization driven by the promoter is relevant with the functions of the encoded genes and is orientation dependent. Expression under P87 was mostly detected in the leaf tip, apical meristem, hydathode, primary root tip and lateral root tip (Figure 4–5) where auxin plays a crucial role during plant development and signaling. Such findings were corroborated with higher expression of *CaM KMT* in the early stages of plant growth (at cotyledon stage) rather than in older leaves and active role of CaM KMT in auxin signaling (J. Banerjee, R. L. Houtz and I. B. Maiti, unpublished data). It has been demonstrated earlier [59] that the methylation level of calmodulin was lower in the apical root segments and young lateral roots compared to the highly differentiated mature tissues. This suggests that in the differentiated tissues most of the calmodulins were already methylated but in the regenerating tissues the methylation of calmodulin was continued due to the P87 activity. The expression under P85 was stronger in the older leaves and root tissues; especially in the midrib or stele part with significant expression in trichomes (Figure 4–5). Our results corroborate the earlier findings that the senescence associated gene (At4g35985) is expressed strongly in older leaves compared to young leaves and since the gene is involved in senescence signaling, a significant root expression is also expected [60]. In addition TAIR gene expression data (Genevestigator) matched our experimental results, showing that the expression of At4g35985 is higher in root tissues compared to older leaves (Figure S6). It has been reported that transport of cytokinins from root to shoot is involved in senescence signaling [61]. Another study reported that a large percentage of genes (40%) are induced in rice roots under Fe deficiency, and these were classified as senescence related [60]. Hence At4g35985 might be involved in senescence signaling having its maximum expression in roots. The differential expression of P85 and P87 using GFP and GUS reporter genes, respectively, clearly documented that the root expression under P85 is stronger compared to the expression under P87 (Figure 4D–E compared to Figure 4J–K and Figure 5G–H compared to 5D) which corroborates the TAIR Genevestigator expression analysis (Figure S6). A recent study reported that the trichomes development is related to the ectopic expression of Capsicum-specific cell wall protein (*Capsicum annuum* senescence-delaying 1 gene) in *N. benthamiana* and the *Capsicum annuum* senescence-delaying 1 gene is strongly expressed in roots [62]. A number of studies have been done on WRKY transcription factors and those are mostly associated with pathogen defense, wounding, trichome development, and senescence [63]. In the present study, the P85–P87 promoter region possesses a number of WRKY transcription factor binding sites (Table 1); these *cis*-sequences and related transcription factors may regulate the senescence as well as trichome development, further studies are needed to evaluate the exact function of these *cis*-elements.

Interestingly the P85–P87 bidirectional promoter showed flower as well as stamen specific expression in both orientations in *Arabidopsis* as well as tobacco system (Figure 4–5 and Figure 8). A number of pollen specific *cis-*elements (13 POLLEN1LELAT52; AGAAA; 9 *cis*-elements responsible for late pollen development and pectate lyase activity, GTGA-motif) are distributed throughout the promoter region (Table 1). The involvement of a conserved GTGA motif in pollen specific expression has been reported in the promoter of tobacco late pollen gene g10 and tomato lat56 promoter [64]. In maize, the upstream region of *ZmMADS2* gene holds AGAAA and GTGA motifs in a number of clusters [65] and MADS box transcription factors are necessary for anther and pollen maturation. Additionally, tomato endo β-mannanase, associated with anther and pollen development contains four AGAAA motifs in its upstream region [66]. In the present study, we speculate that the binding of transcription factors in those *cis-*elements throughout the bidirectional promoter region might be responsible for its significant floral expression at both directions.

To elucidate the importance of different *cis*-regulatory elements, 12 different deletion constructs from the 3′-end of the P87 promoter were generated and assayed in a transient protoplast system. A 100 bp 3′-end deletion resulted in about 40% reduction in activity compared to the whole promoter fragment and further deletions did not show any significant changes in activity (Figure 7). The 100 bp region was found to have one as-1 element (TGACG) in this region. As the as-element is responsible in regulating oxidative stress signaling pathway [67], [68], the as-1 element in the P85–P87 promoter might be crucial for the involvement of the adjacent genes to various stress. Our further studies confirmed that the promoter was active in tobacco in both directions almost in an identical fashion to *Arabidopsis*. However, GUS expression in tobacco pollen grains was found to vary from stronger to moderate and that might be due to the difference in their developmental stages, which warrants further study.

The IR was found to possess a number of light responsive elements (Table 1) like G-box (CACGTC) and that might be responsible for senescence as corroborated from the significant expression under P85 in older leaves. The role of G-box binding factor in the onset of *Arabidopsis* leaf senescence has been reported earlier [69]. Another study documented that in the poplar *Rbcs* gene promoter, a number of light responsive transcription factor binding sites (ATCT-motif, Box I, GAG-motif, I-box, G-box, BoxII, GATA-motif, and TCT-motif) were available and the promoter was mostly active in green tissues [70]. Interestingly the bidirectional promoter reported in the present study also showed green tissue specific expression in both directions probably due to the presence of different light responsive elements (Table 1). On the other hand, the root motif (ATATT) characterized by earlier researchers was found to be responsible for the promoter expression in the elongation zone and the vasculature part in roots [71], [72]. The present bidirectional promoter also possesses a number of similar transcription factor binding sites for root specific expression and that might be responsible for strong expression in both orientations in root tip and stele regions.

The bidirectional promoter was found to show expressional up-regulation under varying concentrations of NaCl treatments. In both directions, the promoter showed at least 2-fold up-regulation in GUS activities in endogenous condition (Figure 3). Histological staining revealed that in P87::GUS plants, 150 mM NaCl induced more GUS protein localization in regenerating tissues of *Arabidopsis* and tobacco seedlings; whereas in P85::GUS *Arabidopsis* plants, NaCl treatment showed maximum expressional changes in stem and floral tissues (Figure 9). This could be due to the combinatorial effect of different tissue specific and stress related transcription factor binding with the available *cis*-elements in the promoter fragment. In addition, the bidirectional promoter showed up-regulation in promoter activity after biotic stress (*P. tabacina* inoculation) as revealed by GUS histochemical staining and GUS enzymatic activity (Figure 10) in transgenic GFP::P85–P87::GUS tobacco plant leaves. The IR containing four GT-1 box (GAAAAA) (Table 1) elements might be responsible for NaCl responsiveness and biotic stress inducibility. The GT-1 box in the soybean calmodulin isoform (*SCaM-4*) promoter was found to be recognized by GT-1 like transcription factor leading to NaCl and pathogen induced gene expression in *Arabidopsis* and soybean [73]. Similarly an artificially designed bidirectional promoter having a GT-1 box showed expressional up-regulation under NaCl, salicylic acid and IAA treatment in both orientations [74]. Eukaryotic enhancer region is known to possess the ability to function in an orientation independent fashion [75], however other factors might be responsible for the promoter activity in an orientation dependent manner which is yet to be established. Hence it can be concluded that different *cis*-elements may be responsible for the expression of the bidirectional promoter throughout the vegetative as well as reproductive tissues in plants.

Promoters are one of the most vulnerable parts of genetic circuits which can undergo a loss of function due to naturally occurring mutations, insertions or deletions. Hence, to enhance the evolutionary stability of a genetic circuit protection of the promoter is crucial [76]. Engineered genetic elements sometimes lose their functions in host systems due to selection pressure and extra metabolic load in host systems [76]–[78]. In nature, many organisms co-regulate the expression of multiple genes in opposite directions, which are involved in similar functions and pathways [8], [16], [79]. The co-regulatory functions of natural bidirectional promoters are achieved by the control of different regulatory elements in opposite strands of the promoter [80]. Similarly in the present study the bidirectional promoter was found to show expressional up-regulation under salt stress in both the orientations. Therefore, it can be concluded that the bidirectional promoter used in the present study share regulatory elements that regulate expression in opposite orientations and due to its natural origin, is expected to survive better in the host cells against selection pressure and metabolic load compared to other synthetic promoters. Further isolation and characterization of natural bidirectional promoters might be a useful tool for gene manipulation in modern agricultural biotechnology and gene pyramiding.

Bidirectional promoters are more efficient compared to the unidirectional promoter for biotechnological improvement through regulating a number of genes. Tissue specific and stress inducible expression of this bidirectional module might be useful for deciphering the possible functional role of two adjacent genes from Arabidopsis. The bidirectional promoter analyzed in this study is of interest due to its differential tissue specific expression and its variation in expressional magnitude in both orientations. In one direction (At4g35987) it is very active in shoot tip, root tip and apical meristemic region whereas in other orientation (At4g35985) it is strong in mature tissues. Hence for developing a broad spectrum fungal or bacterial disease resistance multiple genes could be simultaneously expressed in both orientations to obtain resistance from juvenile stage to the mature stage of plants in root, leaf as well as floral tissues. Regulatory elements of bidirectional promoters showing tissue specific and stress inducible promoter-functions in heterologous systems will be useful to generate hybrid promoters for various biotechnological applications in plants. Furthermore, bidirectional promoters could be useful in gene stacking where multiple genes are expressed in transgenic plants and in molecular farming for the production of vaccines, pharmaceuticals, and plastics.

## Supporting Information

Figure S1 **Transient expression of P85–P87 bidirectional promoter in onion epidermal cells.** **A. GFP expression analysis** Superimposed (bright field and green fluorescent), fluorescent and bright field images of onion epidermal cells bombarded with respective promoter construct DNA loaded gold particles are presented. Control represents untransformed onion epidermal cell visualized under CLSM. **B. GUS expression analysis** Light microscopy images of X-gluc treated onion epidermal cells bombarded with respective promoter construct DNA loaded gold particles are presented. Control represents untransformed onion epidermal cells treated with X-gluc.
(TIFF)

Figure S2 **Confocal laser scanning microscopic analysis of empty vector control Arabidopsis seedlings at different growth stages.** No detectable GFP fluorescence was observed in periphery of cotyledon in five-day old Arabidopsis seedling (A) and in leaf tip (B), apical meristemic region (C), primary root (D) and lateral root (E) of three-week-old plants. Green fluorescence image (left); bright field image (middle), superimposed image (right) are shown. Bar 250 µm in each image.
(TIFF)

Figure S3 **Confocal laser scanning microscopic analysis of reproductive tissues of empty vector control Arabidopsis plants.** Negligible autofluorescence in anther and stigma of flower (A) and no detectable fluorescence in the tip of silique (B) base of silique (C) are visualized. Green fluorescence image (left); bright field image (middle), superimposed image (right) are shown. Bar 250 µm in each image.
(TIFF)

Figure S4 **Confocal laser scanning microscopic analysis of tobacco empty vector control plants.** No detectable GFP fluorescence was observed in leaf (A) and root (B) tissues of 21-day-old tobacco seedlings. Green fluorescence image (left); bright field image (middle), superimposed image (right) are shown. Bar 250 µm in each image.
(TIFF)

Figure S5 **The 1258** **bp bidirectional promoter sequence located between** ***At4g35985*** **and** ***At4g35987*** **in head-to-head orientation.** The upstream nucleotide of the start codon of *At4g35985* (CAT in red) is designated as position 1 and the upstream nucleotide of the start codon of *At4g35987* (ATG in red) is designated as position 1258. The major 5′-untranslated region (UTR) for *At4g35985* was up to nucleotide G which is 33 bp upstream from corresponding start codon (shown as green). The TATA-box (TATAAA) was located in negative strand (brown) which is 30 bp upstream of major transcription start site (TSS) of *At4g35985*. The 5′-UTR for *At4g35987* was up to nucleotide G which is 93 bp upstream from corresponding start codon (shown as underline) and a CAAT-box (orange) is located 74 bp upstream from TSS of *At4g35987*.
(TIFF)

Figure S6 **Expression analysis of the** ***Arabidopsis*** **senescence associated gene (** ***At4g35985*** **) from public database.** The data was collected from Genevestigator expression analysis using TAIR website (http://www.arabidopsis.org/).
(TIFF)

Methods S1 **Transient expression of P85–P87 bidirectional promoter in onion epidermal cells using Gene gun.**
(DOC)
