# Supplementary figures and images for: An Intergenic Region Shared by At4g35985 and At4g35987 in Arabidopsis thaliana Is a Tissue Specific and Stress Inducible Bidirectional Promoter Analyzed in Transgenic Arabidopsis and Tobacco Plants
Source: PLoS One. 2013 Nov 19;8(11):e79622. doi: 10.1371/journal.pone.0079622 (PMC3834115; doi:10.1371/journal.pone.0079622)

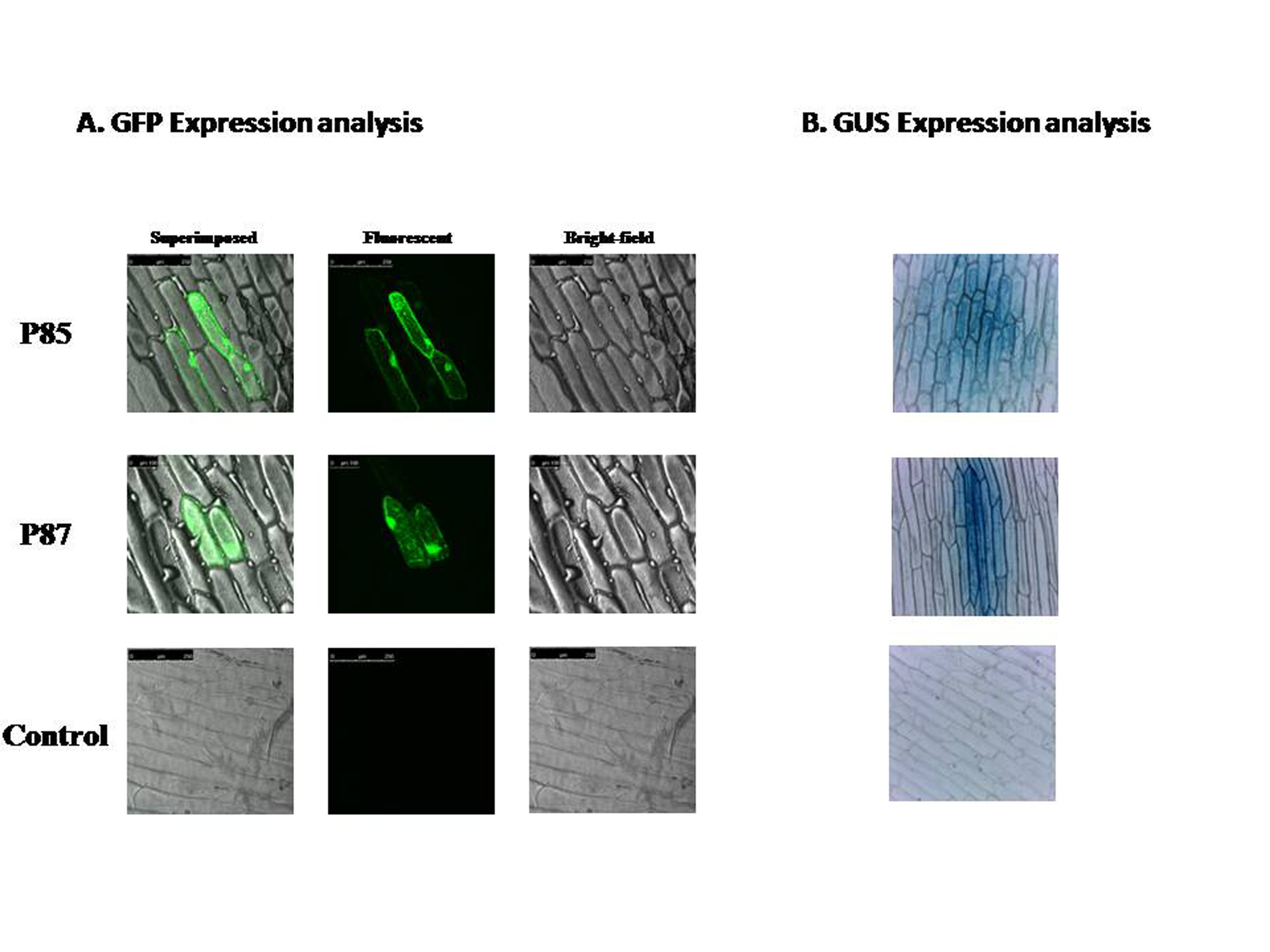

Supplement: Figure S1 — Transient expression of P85–P87 bidirectional promoter in onion epidermal cells. A. GFP expression analysis Superimposed (bright field and green fluorescent), fluorescent and bright field images of onion epidermal cells bombarded with respective promoter construct DNA loaded gold particles are presented. Control represents untransformed onion epidermal cell visualized under CLSM. B. GUS expression analysis Light microscopy images of X-gluc treated onion epidermal cells bombarded with respective promoter construct DNA loaded gold particles are presented. Control represents untransformed onion epidermal cells treated with X-gluc. (TIFF) [file pone.0079622.s001.tiff]

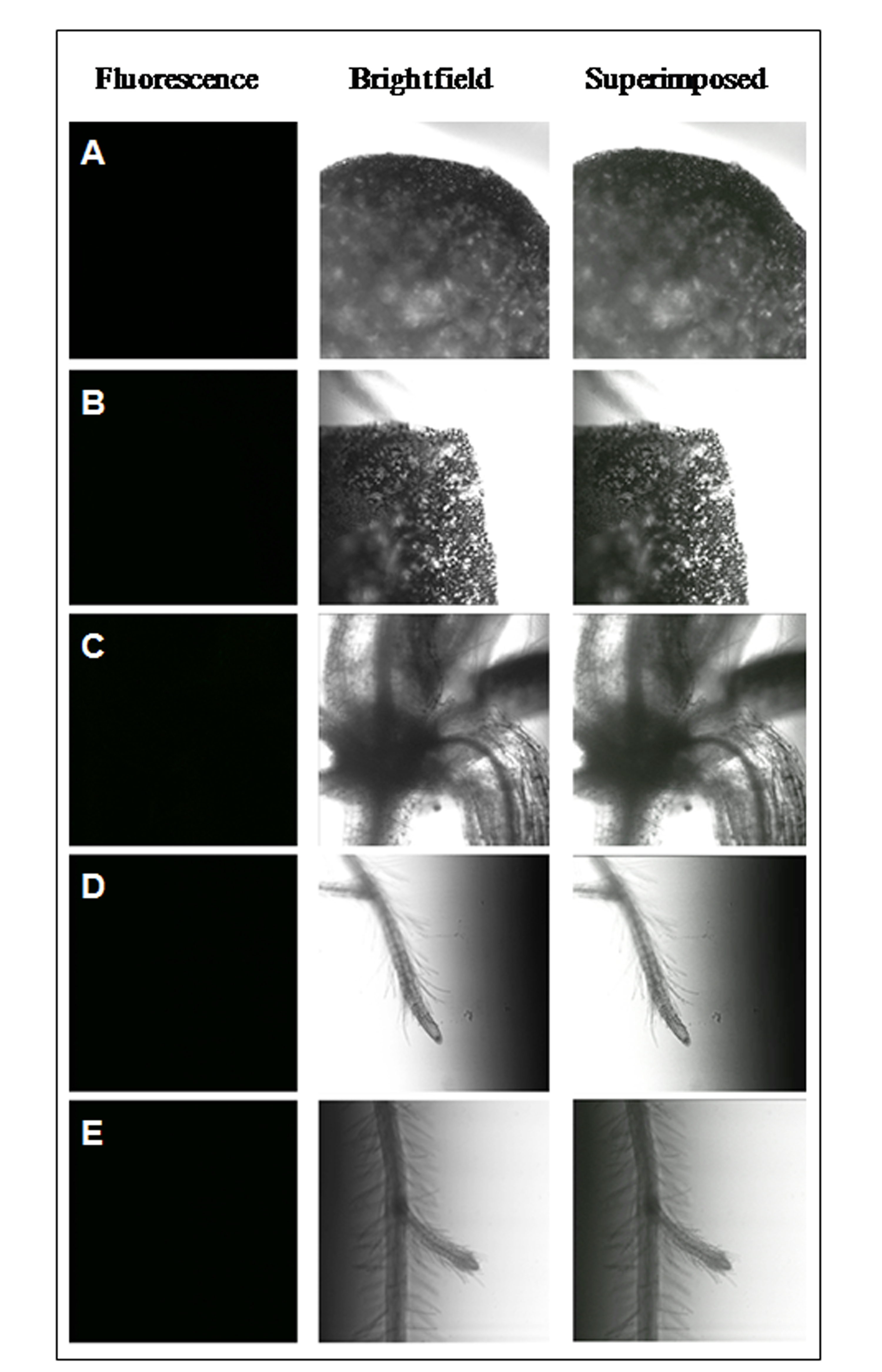

Supplement: Figure S2 — Confocal laser scanning microscopic analysis of empty vector control Arabidopsis seedlings at different growth stages. No detectable GFP fluorescence was observed in periphery of cotyledon in five-day old Arabidopsis seedling (A) and in leaf tip (B), apical meristemic region (C), primary root (D) and lateral root (E) of three-week-old plants. Green fluorescence image (left); bright field image (middle), superimposed image (right) are shown. Bar 250 µm in each image. (TIFF) [file pone.0079622.s002.tiff]

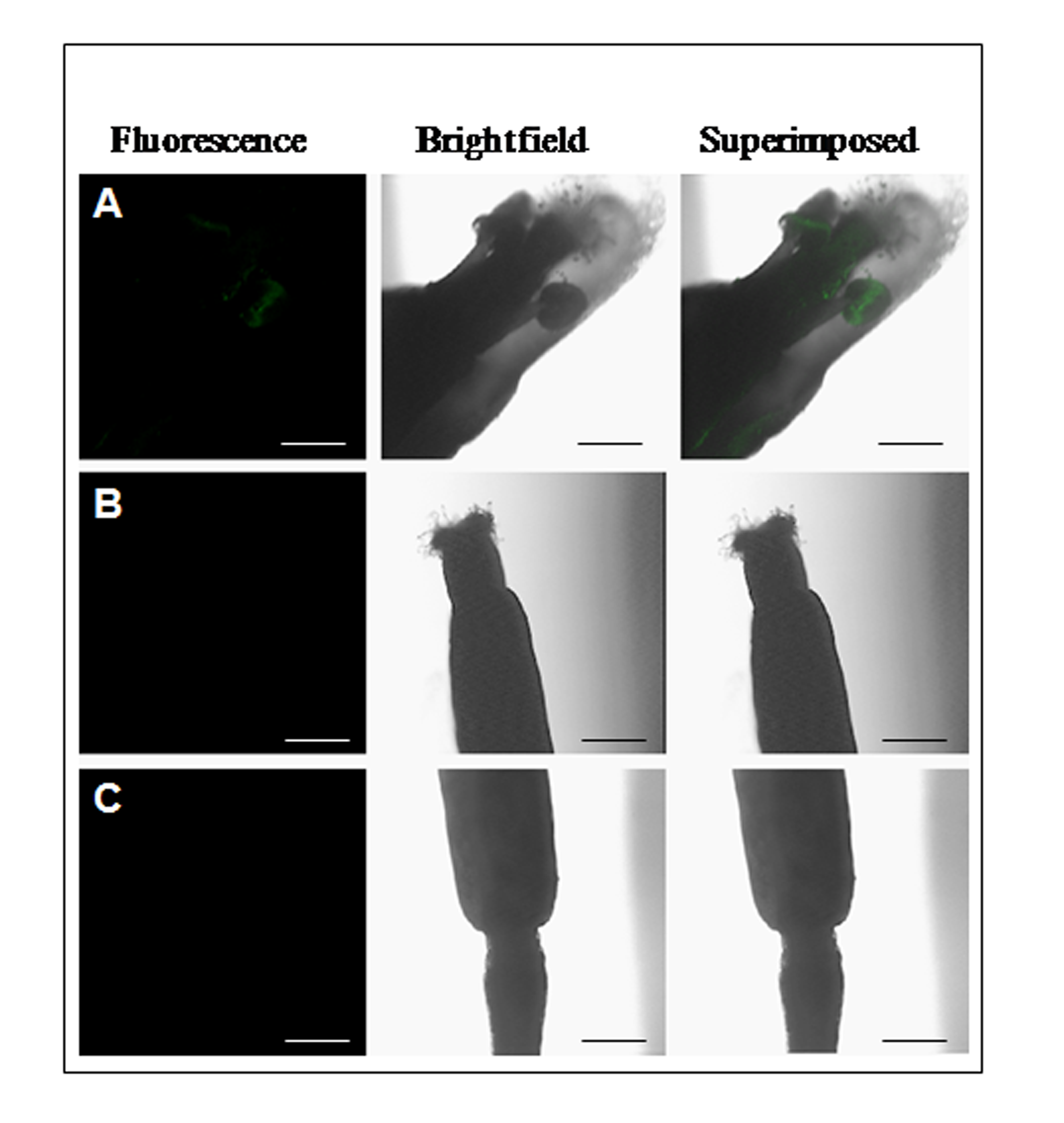

Supplement: Figure S3 — Confocal laser scanning microscopic analysis of reproductive tissues of empty vector control Arabidopsis plants. Negligible autofluorescence in anther and stigma of flower (A) and no detectable fluorescence in the tip of silique (B) base of silique (C) are visualized. Green fluorescence image (left); bright field image (middle), superimposed image (right) are shown. Bar 250 µm in each image. (TIFF) [file pone.0079622.s003.tiff]

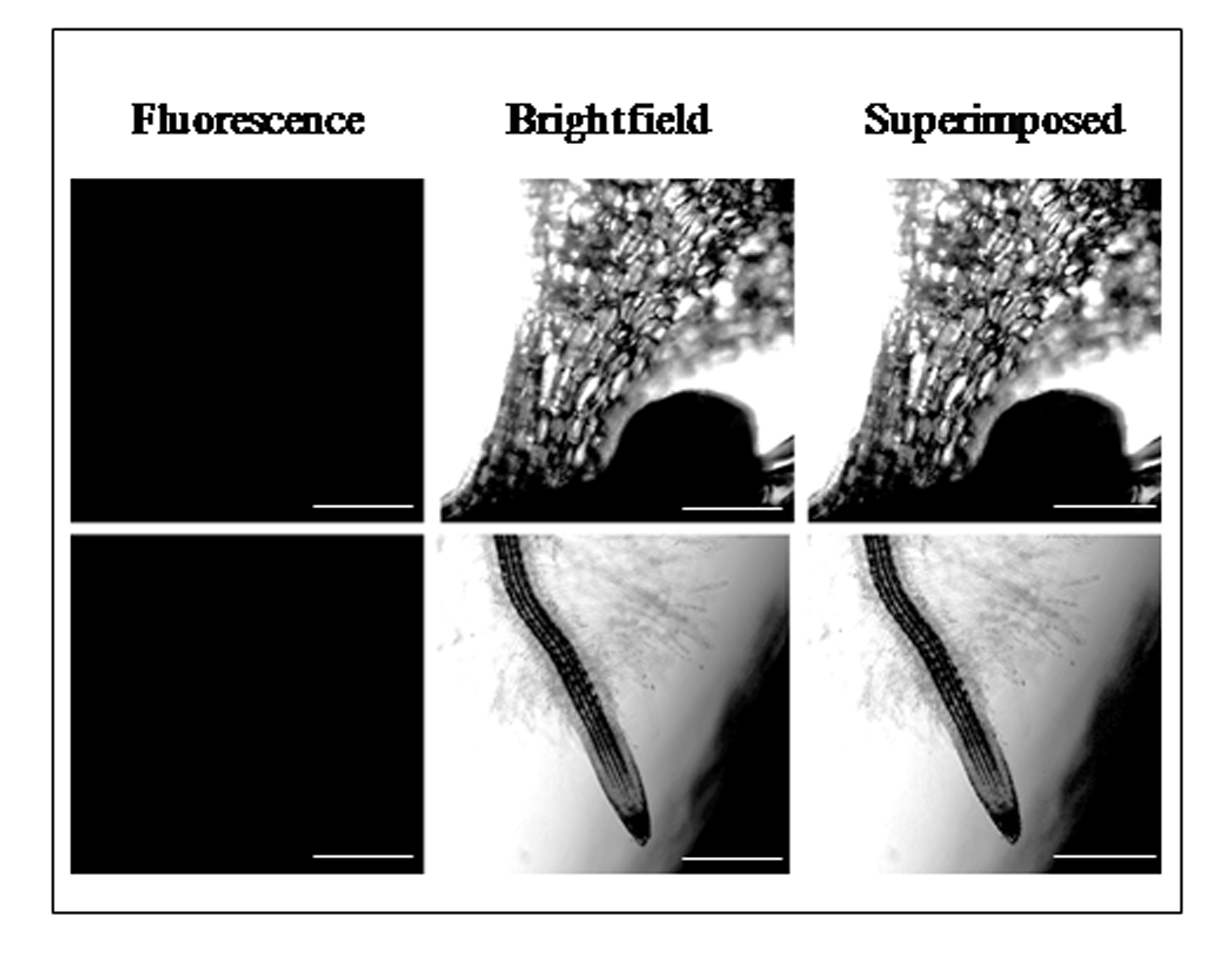

Supplement: Figure S4 — Confocal laser scanning microscopic analysis of tobacco empty vector control plants. No detectable GFP fluorescence was observed in leaf (A) and root (B) tissues of 21-day-old tobacco seedlings. Green fluorescence image (left); bright field image (middle), superimposed image (right) are shown. Bar 250 µm in each image. (TIFF) [file pone.0079622.s004.tiff]

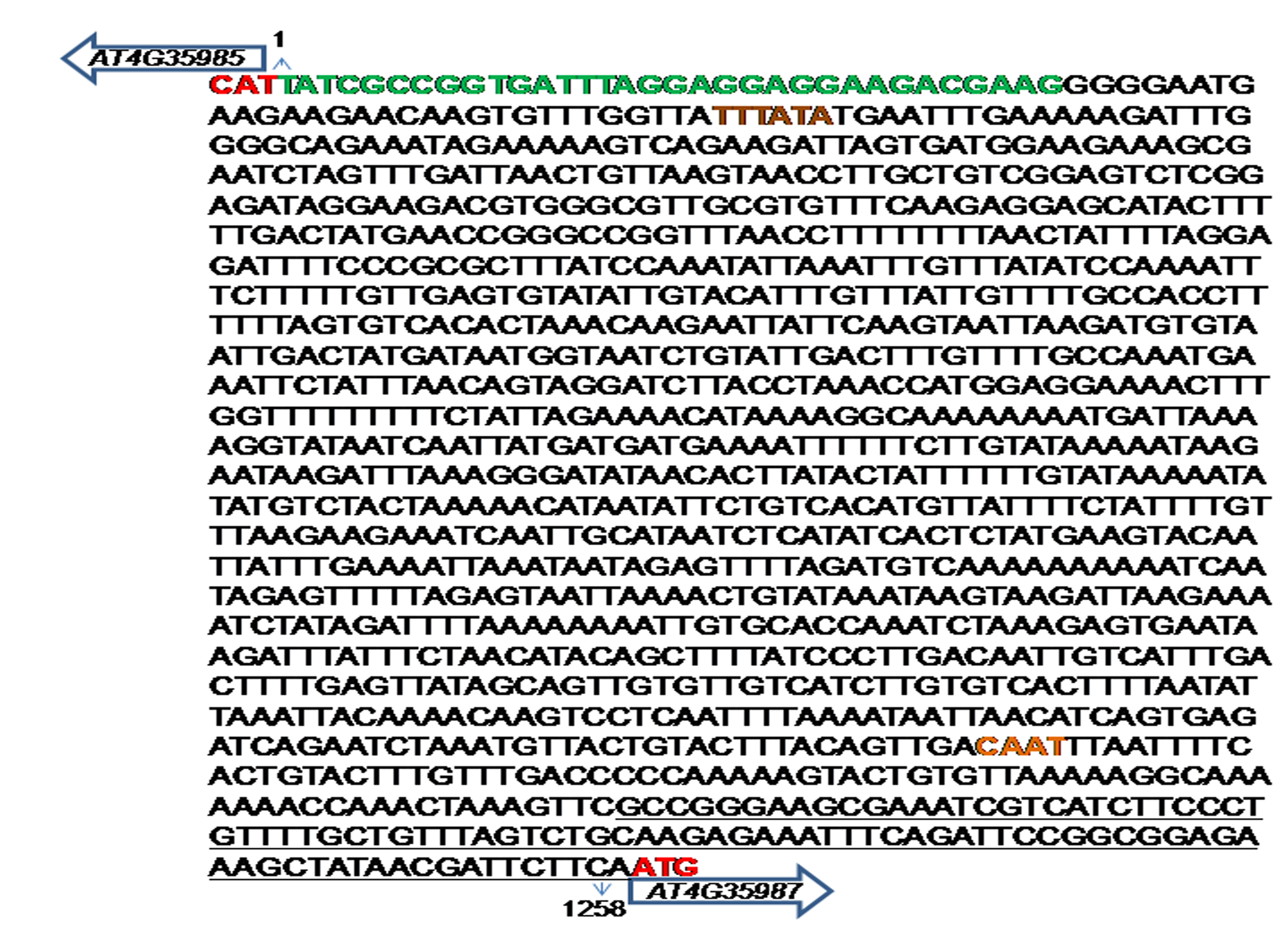

Supplement: Figure S5 — The 1258 bp bidirectional promoter sequence located between At4g35985 and At4g35987 in head-to-head orientation. The upstream nucleotide of the start codon of At4g35985 (CAT in red) is designated as position 1 and the upstream nucleotide of the start codon of At4g35987 (ATG in red) is designated as position 1258. The major 5′-untranslated region (UTR) for At4g35985 was up to nucleotide G which is 33 bp upstream from corresponding start codon (shown as green). The TATA-box (TATAAA) was located in negative strand (brown) which is 30 bp upstream of major transcription start site (TSS) of At4g35985. The 5′-UTR for At4g35987 was up to nucleotide G which is 93 bp upstream from corresponding start codon (shown as underline) and a CAAT-box (orange) is located 74 bp upstream from TSS of At4g35987. (TIFF) [file pone.0079622.s005.tiff]

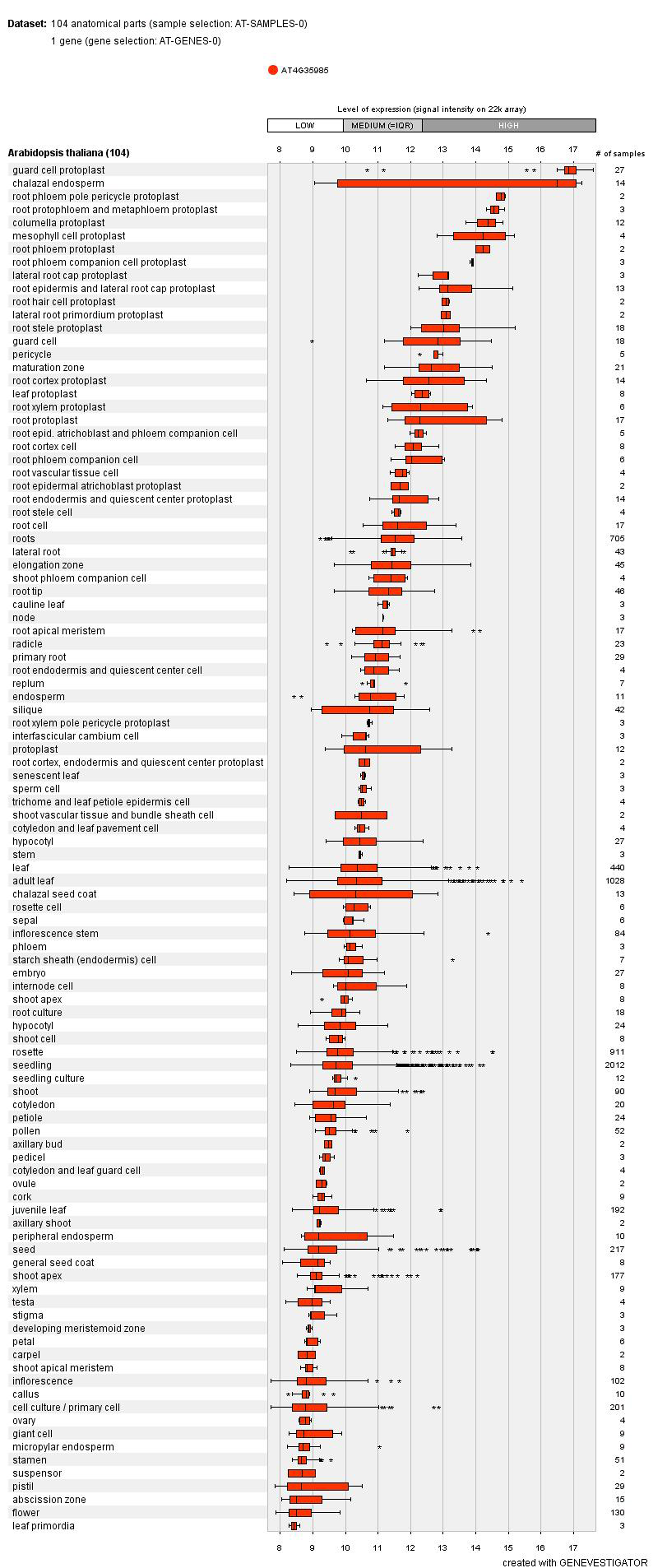

Supplement: Figure S6 — Expression analysis of the Arabidopsis senescence associated gene ( At4g35985 ) from public database. The data was collected from Genevestigator expression analysis using TAIR website (http://www.arabidopsis.org/). (TIFF) [file pone.0079622.s006.tiff]
