# Supplementary material for: An Intergenic Region Shared by At4g35985 and At4g35987 in Arabidopsis thaliana Is a Tissue Specific and Stress Inducible Bidirectional Promoter Analyzed in Transgenic Arabidopsis and Tobacco Plants
Source: PLoS One. 2013 Nov 19;8(11):e79622. doi: 10.1371/journal.pone.0079622 (PMC3834115; doi:10.1371/journal.pone.0079622)
Supplement: Methods S1 — Transient expression of P85–P87 bidirectional promoter in onion epidermal cells using Gene gun. (DOC) [file pone.0079622.s007.doc]

**Gene gun experiment**

The inner epidermal cell layers of onion were placed on a petri-dish containing MS media. Gold micro-carrier coated with DNA of promoter constructs P85 and P87 were prepared separately according to manufacturer’s protocol (Biorad). Briefly, an aliquot of 50µg of purified plasmid was precipitated onto 25 mg of 1µ gold particles using 100µl of 0.05 M spermidine in presence of 100µl of 1 M CaCl_2_ and finally loaded particles were used to bombard onion epidermal cells at 200 psi. The bombarded onion epidermal cells were incubated for 48 h at 25ºC. GFP expression analysis was carried out using a Confocal laser scanning microscopy (CLSM) at λex/λem 488/509nm. GUS expression analysis was performed by staining samples with X-Gluc. Photographs of X-gluc treated samples were captured using a Leica light microscope at 10X magnification.
